# Effective mismatch repair depends on timely control of PCNA retention on DNA by the Elg1 complex

**DOI:** 10.1093/nar/gkz441

**Published:** 2019-05-22

**Authors:** Lovely Jael Paul Solomon Devakumar, Christl Gaubitz, Victoria Lundblad, Brian A Kelch, Takashi Kubota

**Affiliations:** 1 Institute of Medical Sciences, School of Medicine, Medical Sciences & Nutrition, University of Aberdeen, Foresterhill, Aberdeen AB25 2ZD, Scotland, UK; 2 Department of Biochemistry and Molecular Pharmacology, University of Massachusetts Medical School, Worcester, MA 01605, USA; 3 Salk Institute for Biological Studies, La Jolla, CA 92037-1099, USA

## Abstract

Proliferating cell nuclear antigen (PCNA) is a sliding clamp that acts as a central co-ordinator for mismatch repair (MMR) as well as DNA replication. Loss of Elg1, the major subunit of the PCNA unloader complex, causes over-accumulation of PCNA on DNA and also increases mutation rate, but it has been unclear if the two effects are linked. Here we show that timely removal of PCNA from DNA by the Elg1 complex is important to prevent mutations. Although premature unloading of PCNA generally increases mutation rate, the mutator phenotype of *elg1*Δ is attenuated by PCNA mutants PCNA-R14E and PCNA-D150E that spontaneously fall off DNA. In contrast, the *elg1*Δ mutator phenotype is exacerbated by PCNA mutants that accumulate on DNA due to enhanced electrostatic PCNA–DNA interactions. Epistasis analysis suggests that PCNA over-accumulation on DNA interferes with both MMR and MMR-independent process(es). In *elg1*Δ, over-retained PCNA hyper-recruits the Msh2–Msh6 mismatch recognition complex through its PCNA-interacting peptide motif, causing accumulation of MMR intermediates. Our results suggest that PCNA retention controlled by the Elg1 complex is critical for efficient MMR: PCNA needs to be on DNA long enough to enable MMR, but if it is retained too long it interferes with downstream repair steps.

## INTRODUCTION

Maintenance of genome stability requires accurate replication of DNA coupled with constant surveillance by the repair machinery. Proliferating cell nuclear antigen (PCNA) acts as a central co-ordinator for DNA repair as well as DNA replication (1). PCNA is important for mismatch repair (MMR) and required at multiple steps during the MMR process (2–5). However, how PCNA regulation affects MMR is not fully understood. In particular, it is unknown if PCNA residence time on DNA is important for MMR.

PCNA is a ring-shaped homotrimeric complex that encircles DNA to act as a sliding clamp, ensuring processivity of DNA polymerases. It also operates as a platform for recruitment of numerous other proteins involved in DNA replication, DNA damage repair, mismatch repair, and chromatin structure and assembly (1). PCNA levels on DNA are regulated by loading and unloading. During DNA replication, the hetero-pentameric Replication Factor C (RFC) complex, composed of largest subunit Rfc1 and smaller subunits Rfc2, 3, 4 and 5, loads PCNA at primer-template junctions as synthesis of each Okazaki fragment initiates (6–8). After completion of each Okazaki fragment, the Elg1 RFC-like complex (Elg1-RLC), which comprises the Elg1 subunit associated with the Rfc2–5 subunits, functions to unload PCNA from the lagging strand (9–12). In the absence of Elg1, PCNA over-accumulates on recently replicated chromatin in the wake of replication forks (11). The role of the Elg1-RLC in PCNA unloading appears to be conserved in humans, since ATAD5 (the mammalian ortholog of Elg1) is required for proper removal of PCNA from chromatin in human cell lines (13,14).

Elg1 is critical for genome maintenance. In budding yeast, loss of the *ELG1* gene causes gross chromosomal rearrangements, increased sister chromatid recombination, defective sister chromatid cohesion, derailed telomere length maintenance and sensitivity to the DNA alkylating drug methyl methanesulfonate (MMS) (15–21). The requirement for Elg1 in genome maintenance is conserved in higher eukaryotes, since mice with reduced expression of ATAD5 exhibit genome instability and have a high tumor incidence (22). In humans, somatic mutations in *ATAD5* have been found in primary endometrial tumors, and *ATAD5* was identified as a susceptibility locus for invasive epithelial ovarian cancer (22,23). PCNA over-accumulation on DNA is the main cause of genome instability observed in yeast cells lacking Elg1 (24,25). In addition to the defects described above, yeast cells lacking Elg1 exhibit increased mutation rates (26), suggesting that Elg1 is required for suppressing spontaneous mutations. However, it is unknown if the increase in mutation rate in *elg1Δ* is the consequence of over-accumulation of PCNA on DNA, and whether it arises from defective MMR.

The MMR pathway corrects mispaired bases resulting from replication errors (2–5). Defects in MMR cause increased mutation rates and result in the development of different cancers including Lynch syndrome (27). In eukaryotic MMR, mispaired bases are recognized by two partially redundant heterodimers of MutS-related proteins, Msh2–Msh6 and Msh2–Msh3 (2–5). The Msh2–Msh6 complex primarily recognizes base-base mispairs and small insertion/deletion mispairs, whereas the Msh2–Msh3 complex tends to recognize larger insertions/deletions as well as some single base mispairs (28). After binding to a mismatch and adenosine triphosphate, these MutS homologs are converted into closed clamp forms that trap DNA and can slide along it (29,30). The MutS homolog then recruits a MutL endonuclease homolog complex (Mlh1–Pms1 or Mlh1–Mlh3 *in Saccharomyces cerevisiae*), targeting repair to the newly synthesized DNA strand along with accessory factors including PCNA, RFC, DNA polymerase delta, RPA and exonuclease 1 (Exo1) (2–4,31–33). Two different pathways are proposed to act at the MMR excision step—an ‘Exo1-dependent’ pathway where Exo1 removes mispairs, or an ‘Exo1-independent’ pathway where Mlh1-Pms1 endonuclease cleaves multiple times to remove mispairs (34,35).

PCNA is involved in multiple steps of MMR. Msh3 and Msh6 interact with PCNA through PCNA-interacting peptide (PIP) motifs (31,36–37). Yeast cells expressing Msh3 and Msh6 mutants that lack PIP motifs exhibit substantial reduction of the MMR activity, indicating that the PIP motif plays an important, although not completely essential, role in MMR (31,37). It has been proposed that the PIP motif of Msh6 tethers Msh2–Msh6 to PCNA on replication forks and is important for replication machinery-coupled mispair recognition (38,39). Msh2–Msh6 also inhibits PCNA unloading through its PIP motif, maintaining the post-replicative temporal window for MMR (40). PCNA retained or loaded on DNA induces activation of the human MutL endonuclease homolog in a strand specific, mismatch- and MutS homolog-dependent manner (41). The orientation of PCNA on DNA determines strand specificity of MMR, directing removal of the mispaired bases specifically from the newly synthesized DNA strand (40,41). Many PCNA mutants in yeast exhibit increased mutation rates in the absence of Exo1, suggesting a central role for PCNA in the Exo1-independent MMR pathway (35). These findings suggest that PCNA coordinates multiple reactions in MMR, but it is not known how PCNA over-retention affects MMR and whether timely PCNA removal is important.

Here, we demonstrate that yeast cells lacking Elg1 exhibit increased mutation rate caused by PCNA over-retention on DNA after DNA replication. In general, premature unloading of PCNA causes increased mutation rates, but dissociation-prone PCNA mutants PCNA-R14E and PCNA-D150E can attenuate the *elg1Δ* mutator phenotype. In contrast, the *elg1*Δ mutator phenotype is exacerbated by retention-prone PCNA mutants that we identify in this study. Epistasis analysis suggests that PCNA over-accumulation interferes with both MMR and MMR-independent process(es). We focused on the effect of PCNA accumulation on MMR, and found that PCNA over-retained behind replication forks over-recruits Msh2–Msh6 to chromatin through the Msh6 PIP-box motif. PCNA over-retained on DNA prevents Msh2–Msh6- and, to a lesser extent, Msh2–Msh3-dependent MMR, and leads to accumulation of MMR intermediates. Our results suggest that control of PCNA retention time by the Elg1 complex is critical for efficient mismatch repair.

## MATERIALS AND METHODS

### Overexpression dominant negative screening

A total of 61 missense mutations were introduced into the *POL30* gene, which was present on a high-copy plasmid and under the control of the constitutive *ADH* promoter (Supplementary Table S1). Individual residues were selected for mutagenesis based on sequence conservation, and focused mainly on charged amino acids since those amino acids were more likely to mediate protein–protein interactions (42). The plasmids for overexpression of PCNA mutants were transformed into wild-type strain or an *elg1*Δ mutant strain, which retain the wild-type copy of *POL30* in the genome. Whole cell extracts (WCE) were prepared from exponentially growing cultures, and PCNA and its SUMOylated forms were detected by Western blot using anti-PCNA antibody.

### Yeast strains

*Saccharomyces cerevisiae* strains used are listed in Supplementary Table S2. Epitope tagging and gene disruption were carried out using standard polymerase chain reaction (PCR)-based gene-insertion methods (43). PCNA point mutants were constructed either by replacing wild-type PCNA with mutant PCNA (24) or by using a CRISPR-Cas9 genome editing system (44). The PIP mutants of *MSH3* and *MSH6*, and a chimera *msh6(3MBD)* were constructed by the CRISPR-Cas9 system. Plasmids and oligonucleotides used for CRISPR-Cas9 genome editing are listed in Supplementary Tables S1 and S3, respectively. For over-expression of Msh2, Msh3 and Msh6, the yeast strains RDKY5964 and CJY1 were transformed with multicopy plasmids carrying Msh2, Msh3 or Msh6 (45). For microscopy studies, the C-terminus of Pms1 was tagged with four tandem copies of green fluorescent protein (GFP) amplified from the pSM1023 plasmid.

### Preparation of whole-cell extracts and chromatin-enriched fractions and western blotting

WCE and chromatin-enriched fractions were prepared as described previously (11). Western blotting and quantification were performed as described previously (46). Antibodies used were mouse monoclonal anti-PCNA (ab70472, Abcam), rabbit polyclonal anti-histone H3 (ab46765, Abcam), mouse monoclonal anti-Flag M2 (F1804, Sigma), mouse monoclonal anti-HA (HA.11 clone 16B12, Covance) and Rat monoclonal anti-GFP (3H9, #029762, Chromotek).

### Fluctuation analysis of mutation rates

Mutation rates were determined using the *lys2–10A* and *hom3–10* frameshift reversion and the *CAN1* inactivation assays, by fluctuation analysis (28,47) and the Ma-Sandri-Sarkar (MSS) maximum-likelihood method (48,49). Each mutation rate was determined using at least 11 independent cultures. Similarly sized colonies grown 2 days at 30°C on YPD plates were transferred to 2–5 ml of liquid YPD and further incubated overnight. The cells were plated on YPD to count viable cells after appropriate dilution, and SD-Lys or SD-Thr to measure the *lys2–10A* reversion and the *hom3–10* reversion, respectively. For the *CAN1* inactivation assay, the cells were plated on SD-Arg to count the viable colonies after appropriate dilution and SD-Arg + 60 μg/ml canavanine to measure the inactivation of the *CAN1* gene. Colonies were counted after 3 days. In the *lys2–10A* reversion assay for cells over-expressing Msh2, Msh3, and Msh6, SD-Ura-Trp-Leu plates and liquid medium and SD-Ura-Trp-Leu-Lys plates were used. Since cells over-expressing Msh2, Msh3 and Msh6 grow slowly, those cells were incubated for 3 days on the plates and for 2 days in the liquid medium. The 95% confidence intervals were calculated as described previously (48). Comparisons of mutation rates were evaluated using 95% confidence intervals.

### Purification, crystallization and structure determination of PCNA-D17K and PCNA-D21K

PCNA point mutations D17K and D21K were introduced by site-directed mutagenesis (50) in a modified pET-28 vector that contains an N-terminal 6-His tag and a PreScission Protease™ cleavage site. PCNA mutants D17K and D21K were expressed in *Escherichia coli* and purified as previously described (51). For crystallization, 0.9 μl of protein (between 15 and 20 mg/ml) was mixed with an equal volume of well solution in 24-well hanging drop trays at room temperature. The well solutions used were 50 mM sodium citrate pH 5.3 and 1.7 M (NH_4_)_2_SO_4_ for PCNA-D17K, and 50 mM sodium citrate pH 5.6, 2 M (NH_4_)_2_SO_4_ for PCNA-D21K. Crystals appeared within 1 day and continued to grow for 5 days. Crystals were briefly swiped through Paratone N cryo-protectant and frozen at 100K in the cryostream. Diffraction data were collected on a Rigaku system with a Saturn 944 CCD detector. Indexing, integration and scaling was performed with HKL3000 (52). The structure was solved by molecular replacement using PHASER (53) with wild-type PCNA as the search model (PDB ID: 4YHR) (54). Refinement and model building were carried out using phenix.refine (55) and Coot (56). The atomic coordinates of PCNA-D17K and PCNA-D21K were deposited to the protein databank (PDB ID: 6D0R and 6D0Q, respectively). The recent cocrystal of human PCNA bound to a duplex of DNA (PDB ID: 5L7C) (57) was used to model the presence of DNA in the PCNA ring. The wild-type yeast PCNA structure (PDB ID: 4YHR) was superimposed onto human PCNA. The structural model of yeast PCNA bound to duplex DNA (51) was not used for two reasons: (i) the DNA duplex is poorly resolved in the crystal structure, and (ii) the DNA is tilted at too sharp of an angle for proper PCNA sliding.

### Chromatin immunoprecipitation (ChIP)-qPCR

To synchronize replication fork movements between cells, cells were released from a *cdc7–1* block into S phase at 16°C and collected 15 min after release, as performed previously (11). Chromatin immunoprecipitation (ChIP) of Msh6–6HA or PCNA-3myc was performed as described (58) using mouse monoclonal anti-HA (HA.11 clone 16B12, Covance) or mouse monoclonal anti-myc (M047–3, MBL) antibody, respectively. ChIP and corresponding input samples were analyzed by LightCycler 480 II (Roche) using Light cycler SYBR Green master reagent (Roche). ChIP efficiency at each locus was calculated as the mean of three technical replicates.

### Cell imaging

Exponentially growing cells were washed and immobilized on Concanavalin A (C-2631, Sigma) coated μ-Slide (chambered coverslip) with 8 wells (80826, ibidi). The cells are covered with Synthetic complete media and imaged on a Deltavision (Applied Precision) microscope with 100 × 1.35NA objective. Sixteen 0.3 μm z-sections were acquired and image processing and foci count were performed using imageJ.

## RESULTS

### Increased mutation rate in *elg1*Δ is dependent on PCNA accumulation on DNA

**Figure 1.**
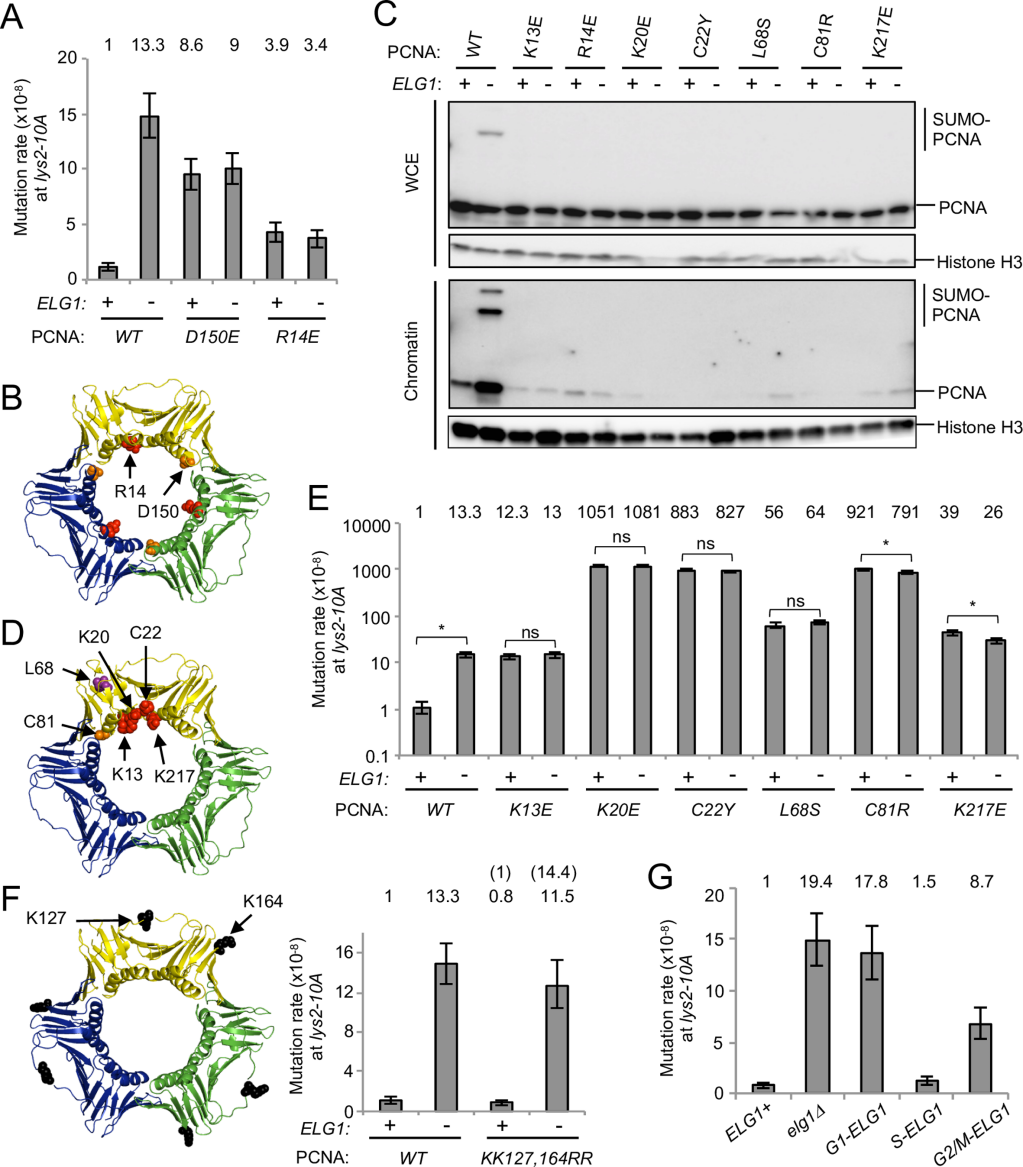
Increased mutation rate in *elg1*Δ is dependent on over-accumulation of PCNA, not SUMOylated PCNA, on DNA after DNA replication. (**A**) Mutation rate analysis was performed to measure the reversion rate of *lys2–10A* to LYS+ phenotype. Fold increases over wild-type are shown above the histogram. Error bars, 95% confidence intervals. (**B**) Structure of the PCNA trimer (PDB ID: 3K4X) (51). The positions mutated are highlighted. (**C**) The inner ring surface PCNA mutants (K13E, R14E, K20E, C22Y and K217E) and the trimer instability PCNA mutants (L68S and C81R) do not accumulate on chromatin in *elg1*Δ. WCE and chromatin-enriched fractions (Chromatin) were prepared from cells expressing PCNA mutants in log phase. The PCNA mutants are the only copy of PCNA in these cells. PCNA and histone H3 (loading control) were detected by Western blotting. (**D**) Structure of the PCNA trimer (PDB ID: 3K4X) (51). The positions mutated are highlighted. (**E**) Mutation rates of *ELG1*^+^ and *elg1*Δ in the wild-type and dissociation-prone PCNA mutants backgrounds at the *lys2–10A* locus. Fold increases over wild-type are shown above. Error bars, 95% confidence intervals. *, no overlapping of error bars; ns, overlapping of error bars. Mutation rates for *ELG1*^+^ and *elg1*Δ in wild-type PCNA background are as shown in panel A, repeated for ease of reference here and in subsequent Figures 1F, 2D and 3B. (**F**) Structure of the PCNA trimer (PDB ID: 3K4X) (51). The positions mutated are highlighted. Mutation rate of *ELG1*^+^ and *elg1*Δ in the wild-type and unSUMOylatable PCNA mutants backgrounds at the *lys2–10A* locus. Fold increase over wild-type is shown above the histogram. Error bars, 95% confidence intervals. (**G**) Mutation rates of the cell-cycle-regulated alleles of *ELG1* (24) at the *lys2–10A* locus. Fold increases over *ELG1*^+^ are shown above. Error bars, 95% confidence intervals.

PCNA accumulation on DNA in *elg1*Δ causes genome instability, including increased recombination of sister chromatids and direct repeats, and elongation of telomeres (24,25). It has been suggested that loss of *ELG1* also results in elevated mutation rate (26). We first tested if PCNA accumulation on DNA causes an elevated mutation rate in *elg1*Δ. To analyze mutation rate, we measured the reversion of +1 frameshift mutations in the *LYS2* (*lys2–10A*) and the *HOM3* genes (*hom3–10*) (28,47). Consistent with a previous report (26), loss of *ELG1* resulted in elevated reversion rates in the Lys+ and Hom+ assays (Figure 1A; Supplementary Figure S1A and Table S4). The *lys2–10A* allele is characterized by a 67-bp ‘InsE’ insertion containing a homonucleotide run of 10 adenine nucleotides at the position 3015 nt (Supplementary Figure S1B). Sequencing the *LYS2* gene of the revertants arising in wild-type and *elg1*Δ confirmed that all reversion events tested involved the deletion of one nucleotide, mainly a single A in the run of 10 As, to restore the correct open reading frame (Supplementary Figure S1B). Reversion was not due to pop-out recombination between 6-bp short repeats that flank the InsE element. To test if the increase in reversion rate in *elg1*Δ is dependent on PCNA accumulation on DNA, we utilized the disassembly-prone PCNA mutant *pol30-D150E* (Figure 1A and B). This mutation disrupts the trimer interface, decreasing PCNA trimer stability *in vitro* (35), and preventing PCNA accumulation on chromatin *in vivo* even in an *elg1*Δ mutant (as demonstrated by the failure of PCNA-D150E to accumulate on chromatin in *elg1*Δ; (24,35). The *pol30-D150E* mutation itself caused an ∼9-fold increase in reversion rate (Figure 1A, column 3). In the disassembly prone mutant *pol30-D150E* background, loss of *ELG1* did not increase the reversion rate compared to *ELG1*^+^ (Figure 1A, columns 3 and 4), but rather, the disassembly-prone mutant *pol30-D150E* moderately rescued the increased mutation rate observed in *elg1*Δ (Figure 1A, columns 2 and 4). These results support the idea that the increased mutation rate in *elg1*Δ is dependent on PCNA accumulation on DNA.

To see a clearer rescue in reversion rate in *elg1*Δ by disassembly-prone mutants, we sought to identify PCNA mutants that fall off DNA spontaneously but do not show a severe mutator phenotype. To this end, we performed a biochemical version of a previously developed protocol that employed overexpression dominant negative (ODN) screening to identify separation-of-function mutations in telomerase subunits (42,59). Briefly, a set of 61 PCNA point mutants (see ‘Materials and Methods’ section) was over-expressed from a multi-copy plasmid in the presence of endogenous PCNA, and chromatin-bound PCNA was assessed by examining the amount of SUMOylated PCNA present in WCE by western blotting. SUMO-PCNA provides a useful proxy for chromatin-bound PCNA since SUMOylation is DNA dependent (9,60). In the *elg1*Δ background, most over-expressed wild-type PCNA and PCNA mutants were SUMOylated efficiently, but 16 PCNA mutants (K13E, R14E, K20E, C22Y/E, L68S/E, K77E, C81R, R110E, E113K, K117E, E143K, K146E, D150K and K217E) exhibited reduced amounts of their SUMOylated forms in WCE (Supplementary Figure S1C), consistent with the possibility that these mutants fall off DNA spontaneously. It is likely that PCNA with mutations near the trimer interface (K77E, C81R, R110E, E113K, K117E, E143K, K146E and D150K) are disassembly-prone. To examine further the phenotypes caused by the mutations in PCNA, we constructed strains expressing each PCNA mutant as the only copy of the *POL30* gene, expressed from its endogenous locus. To test if the inner ring mutants are dissociation-prone, we constructed *pol30-K13E, pol30-R14E, pol30-K20E, pol30-C22Y* and *pol30-K217E* as well as two known trimer instability mutants *pol30-L68S* and *pol30-C81R* as controls. We found that all seven mutants we tested did exhibit a ‘dissociation-prone’ phenotype, as assessed by amounts of PCNA in chromatin fractions (Figure 1C lower panel and D). Screening among these mutants, most exhibited substantially elevated mutation rates (see the next section and Figure 1E), making them unsuitable for testing whether they could rescue the elevated mutation rate of *elg1*Δ. However the mutant *pol30-R14E*, where R14 locates on the inner ring surface (Figure 1B), by itself only slightly elevated mutation rate (Figure 1A, column 5). Combining *pol30-R14E* with *elg1*Δ largely rescued the elevated mutation rate of the single *elg1*Δ mutant (Figure 1A, column 2 and 6). These results strongly suggest that the increased mutation rate in *elg1*Δ is dependent on PCNA accumulation on DNA.

### Premature unloading of PCNA from DNA causes an increase in mutation rate

In addition to the trimer instability mutants *pol30-L68S* and *pol30-C81R*, several PCNA mutants with changes on the inner ring (K13E, R14E, K20E, C22Y and K217E) showed a dissociation-prone phenotype (Figure 1C and D). However, at least three of them (K13E, C22Y and K217E) were previously shown to form stable trimers *in vitro* (35,61). The phenotypes are consistent with the idea that these inner ring mutants can form stable trimers when not DNA-associated, but cannot be retained on chromatin due to inner ring mutations that potentially disrupt interaction with DNA. As previously reported, the inner ring mutants *pol30-K13E, pol30-C22Y* and *pol30-K217E* as well as the trimer instability mutants *pol30-L68S* and *pol30-C81R* display a mutator phenotype (Figure 1E) (35). All dissociation-prone mutants tested here, including new mutants *pol30-R14E* and *pol30-K20E*, exhibit an elevated mutation rate, suggesting that premature unloading of PCNA from DNA contributes to an increase in mutation rate. In these dissociation-prone mutant backgrounds, deletion of *ELG1* did not cause any further increase in mutation rate (Figure 1A and E), consistent with the idea that the elevated mutation rate observed in *elg1*Δ is dependent on PCNA accumulation on DNA.

### SUMOylation of PCNA is not required for increased mutation rates in *elg1*Δ

PCNA accumulated on DNA in *elg1*Δ becomes hyper-SUMOylated (Figure 1C, lane 2) (9). We tested if SUMOylation of PCNA contributes to the increased mutation rate in *elg1*Δ. In the unSUMOylatable *pol30-KK127,164RR* mutant background, deletion of *ELG1* still increased the mutation rate (Figure 1F). Because this unSUMOylatable PCNA mutant accumulates on DNA in *elg1*Δ (9), these results suggest that accumulation of unmodified PCNA on DNA is sufficient to increase the mutation rate in *elg1*Δ. These results also suggest that the increased mutation rate in *elg1*Δ at the *lys2–10A* locus is not due to error-prone DNA polymerases, which are recruited to DNA via PCNA that is ubiquitinated at K164.

### Timely unloading of PCNA by Elg1-RLC during S phase prevents increase in mutation rate

In the absence of Elg1, PCNA accumulates on DNA in the wake of replication forks during S phase and is retained on DNA even in G2/M phase (11,24). Using cell-cycle-regulated alleles of *ELG1* (24), we tested in which cell-cycle phase PCNA accumulation on DNA causes increased mutation rate. We observe that Elg1 expressed in S phase (*S-ELG1*) almost completely rescues the mutator phenotype of *elg1*Δ (Figure 1G). Elg1 expressed in G2/M phase (*G2/M-ELG1*) exhibits a partial rescue phenotype, but Elg1 expressed in G1 phase (*G1-ELG1)* does not. These results suggest that PCNA retention on DNA after DNA replication increases the mutation rate, and timely unloading of PCNA by Elg1-RLC during S phase is important for limiting mutations.

### Identification of new PCNA mutants that are retained on DNA and increase the mutation rate

**Figure 2.**
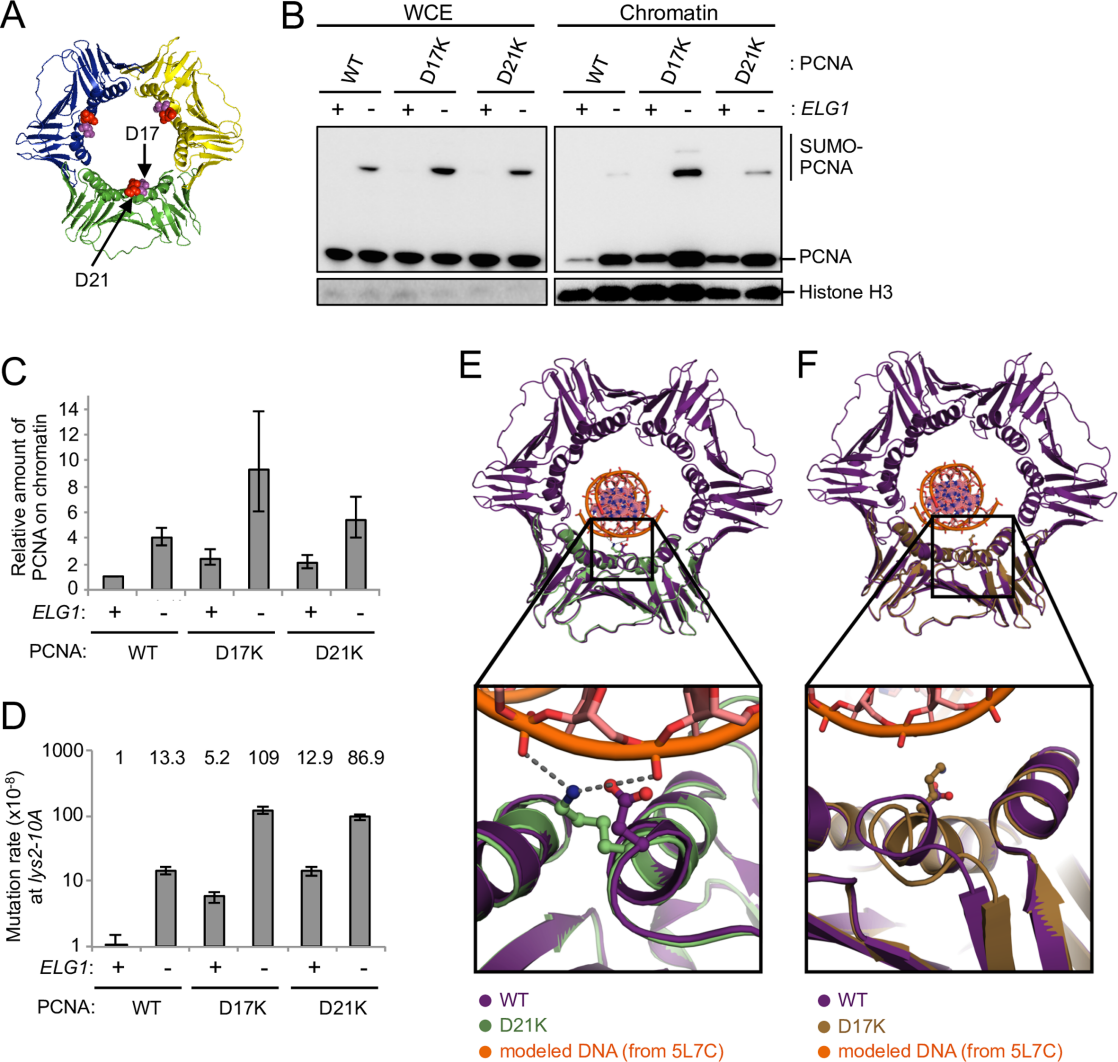
Identification of new PCNA mutants that are retained on DNA due to enhanced electrostatic interaction and cause increase in mutation rate. (**A**) Structure of the PCNA trimer (PDB ID: 3K4X) (51). The positions mutated are highlighted. (**B**) The inner ring surface PCNA mutants (D17K and D21K) accumulate on chromatin. WCE and chromatin-enriched fractions (Chromatin) were prepared from cells expressing PCNA mutants in log phase. The PCNA mutants are the only copy of PCNA in these cells. PCNA and histone H3 (loading control) were detected by Western blotting. (**C**) Quantification of chromatin-bound PCNA and PCNA mutants. Average of two experiments of total PCNA on chromatin (normalized to histone H3), relative to WT, is shown. Error bars, SDs. (**D**) Mutation rates of *ELG1*^+^ and *elg1*Δ in the wild-type and retention-prone PCNA mutants backgrounds at the *lys2–10A* locus. Fold increases over wild-type are shown above. Error bars, 95% confidence intervals. (**E**) 2.8 Å resolution crystal structure of PCNA-D21K (green, PDB ID: 6D0Q) compared with a hybrid model of WT yeast PCNA (purple, PDB ID: 4YHR) with DNA modeled based on a human PCNA:DNA cocrystal structure (PDB ID: 5L7C). Inset shows that lysine 21 could simultaneously interact with two DNA phosphate groups. (**F**) 2.85 Å crystal structure of PCNA-D17K (tan, PDB ID: 6D0R) compared with a hybrid model of WT yeast PCNA (purple, PDB ID: 4YHR) bound to DNA (PDB ID: 5L7C). Inset shows that lysine 17 is positioned within the major groove of DNA, but also induces a conformational change helix 1 and an adjacent loop.

To show that PCNA accumulation on DNA, and not a different effect of *elg1*Δ, is the cause of the increase in mutation rate, we used ODN screening to identify PCNA mutants that are retained on DNA even in the presence of Elg1. Briefly, we selected as candidates mutants that exhibit increased amounts of their SUMOylated forms in WCE when over-expressed, even in the presence of Elg1 (Supplementary Figure S2A and B). We then constructed strains expressing each PCNA mutant as the only copy of the *POL30* gene, expressed from its endogenous locus, and found two PCNA mutants PCNA-D17K and PCNA-D21K that accumulate on DNA even with Elg1 present (Figure 2A–C). PCNA-D21K is unloaded from DNA slowly compared to WT PCNA, and is retained on DNA during S phase and even in G2/M phase (Supplementary Figure S2C). Elg1 can physically interact with PCNA-D17K and actually shows enhanced interaction with PCNA-D21K (Supplementary Figure S2D), suggesting that accumulation of those PCNA mutants on DNA is not due to a loss of interaction with the Elg1-RLC unloader. These retention-prone mutants increase the mutation rate even in the presence of Elg1 (Figure 2D, columns 3 and 5), suggesting that PCNA accumulation on DNA alone and/or the D17K and D21K mutations themselves can increase the mutation rate. Deletion of *ELG1* causes further accumulation of those mutants on DNA (Figure 2B and C). In contrast to the dissociation-prone mutant backgrounds, deletion of *ELG1* further increases the mutation rate in those retention-prone mutant backgrounds (Figure 2D, columns 4 and 6). These results, together with those in Figure 1, suggest that PCNA over-accumulation on DNA, not loss of other functions of Elg1, increases the rate of mutation.

### Structure of the retention-prone PCNA mutants

To determine the molecular mechanism by which these PCNA variants retain their interaction with DNA and Elg1, we determined their structures using x-ray crystallography (Supplemental Table S5). The structures (at 2.80- and 2.85-Å resolution for PCNA-D21K and PCNA-D17K, respectively) reveal that the mutations alter the inner pore of the PCNA ring (Figure 2E and F). In both cases, the PCNA pore becomes more electropositive due to the swap of a negative charge to a positive charge (Supplemental Figure S3). To examine whether the electrostatic changes might strengthen interaction with DNA, we modeled the presence of DNA within the yeast PCNA ring using the recent cocrystal structure of human PCNA bound to a short DNA duplex (57). The D21K mutation positions lysine 21 to interact favorably in a bidentate manner with two backbone phosphates of DNA (Figure 2E). For D17K, lysine 17 is positioned within the major groove of DNA (Figure 2F), where the electrostatic interaction with DNA is also enhanced. Thus, both mutants are expected to have enhanced interactions with DNA, although through slightly differing contacts. For the D21K variant, we observed no significant conformational changes relative to wild-type PCNA (C_α_ RMSD ∼0.48 Å), indicating that the binding of PCNA partner proteins should not be perturbed. The D17K mutation on the other hand disrupts a salt bridge network within the PCNA pore, resulting in partial unraveling of helix 1, and a conformational change of an adjacent loop (residues 20 through 25; Figure 2F). Because this conformational change occurs on the primary interaction face of PCNA, the D17K variant could potentially perturb binding of PCNA partner proteins. To avoid this complication, we focused our further analyses primarily on the D21K variant.

### PCNA over-accumulation on DNA prevents Msh2–Msh6-dependent mismatch repair at the *lys2-10A* locus

**Figure 3.**
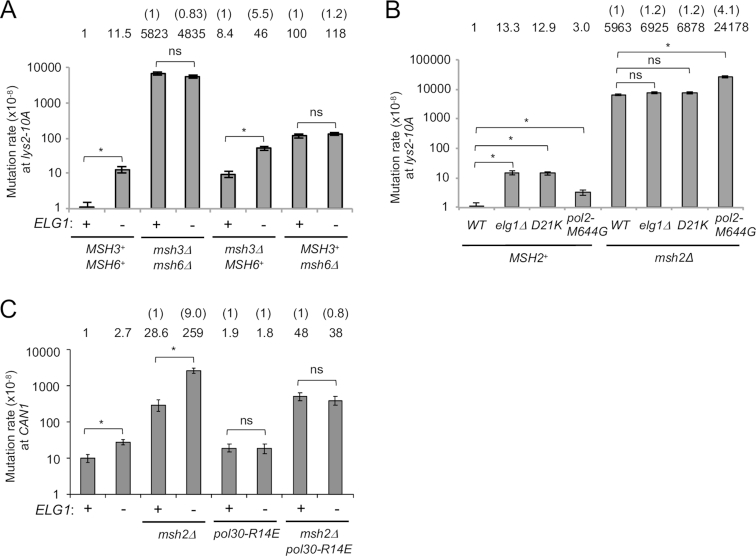
Over-retained PCNA prevents MMR and also affects MMR-independent process(es). (**A**) Mutation rates of *ELG1*^+^ and *elg1*Δ in wild-type, *msh3*Δ and *msh6*Δ backgrounds at the *lys2–10A* locus. Fold increases over wild-type are shown above. The number in the brackets, fold changes over *ELG1*^+^ in each mutant background. Error bars, 95% confidence intervals. *, no overlapping of error bars; ns, overlapping of error bars. (**B**) Mutation rates of *elg1*Δ, *pol30-D21K* and *pol30-M644G* in *MSH2*^+^ and *msh2*Δ backgrounds at the *lys2–10A* locus. Fold increases over wild-type are shown above. The number in the brackets, fold changes over a single *msh2*Δ. Error bars, 95% confidence intervals. *, no overlapping of error bars; ns, overlapping of error bars. (**C**) Mutation rates of *ELG1*^+^ and *elg1*Δ in wild-type, *msh2*Δ, *pol30-R14E* and *msh2*Δ *pol30-R14E* backgrounds at the *CAN1* locus. Fold increases over wild-type are shown above. The number in the brackets, fold changes over *ELG1*^+^ in each mutant background. Error bars, 95% confidence intervals. *, no overlapping of error bars; ns, overlapping of error bars.

We next investigated how PCNA over-accumulation on DNA increases the mutation rate. In particular, we tested whether PCNA over-accumulation on DNA prevents mismatch repair. To this end, we first measured mutation rates in the absence of the mismatch repair proteins Msh3 and Msh6. If PCNA accumulation on DNA increases mutation rate only by preventing mismatch repair, then deletion of *ELG1* will not further increase mutation rate of an *msh3*Δ *msh6*Δ double mutant (which lacks both Msh2–Msh3 and Msh2–Msh6 mismatch recognition complexes). If PCNA accumulation on DNA leads to mutation through other mechanism(s) (e.g. by allowing polymerase slippage and/or preventing DNA polymerase proofreading), then deletion of *ELG1* will synergistically increase the mutation rate with *msh3*Δ *msh6*Δ. An *msh3*Δ *msh6*Δ mutant exhibits an ∼5800-fold increase in mutation rate in the *lys2–10A* assay (Figure 3A). Deletion of *ELG1* did not cause further increase in mutation rate in the *msh3*Δ *msh6*Δ mutant background, indicating that an *elg1*Δ mutant is epistatic with an *msh3*Δ *msh6*Δ mutant (Figure 3A). These results suggest that loss of Elg1 prevents MMR.

We next tested whether deletion of *ELG1* affects Msh2–Msh3 or Msh2–Msh6-mediated repair, or both. In the *lys2–10A* assay, deletion of *ELG1* causes an ∼5.5-fold increase in mutation rate in the *msh3*Δ background where Msh6 is present, but only an ∼1.2-fold increase in the *msh6*Δ background where Msh3 is present (Figure 3A). In the *hom3–10* assay, we observed similar effects (Supplementary Figure S4A). Our results indicate that PCNA accumulation on DNA prevents mainly the Msh2–Msh6-dependent mismatch repair pathway.

Similar to *msh3*Δ *msh6*Δ, combining *elg1*Δ with *msh2*Δ did not cause a synergistic increase in the mutation rate in the *lys2–10A* assay (Figure 3B). In contrast, the *pol2-M644G* mutation exhibits a synergistic effect when combined with *msh2*Δ (Figure 3B and Supplementary Figure S4B) (38). This observation confirms that high mutation rates are measurable using the *lys2–10A* assay, and moreover implies that loss of Elg1 increases mutation rates not by facilitating DNA replication errors (at least, not at the level of errors caused by *pol2-M644G*) but rather by preventing MMR. Consistent with this idea, no synergistic increase in mutation rate was observed on combining the retention-prone mutant *pol30-D21K* with *msh2*Δ (Figure 3B).

When testing mutation rates using the *hom3–10* assay, loss of Elg1 did cause a slight but significant increase in mutation rate in the *msh2*Δ mutant background (Figure 3B; Supplementary Figure S4B and Table S4). This observation leaves open the possibility that loss of Elg1 prevents Msh2-dependent MMR, and simultaneously impacts an additional pathway.

### PCNA over-accumulation on DNA increases mutation rate also through MMR-independent process(es)

We next tested if over-accumulated PCNA also affects MMR at the *CAN1* locus. The *CAN1* inactivation assay measures a broader spectrum of mutation types (including small and large insertion/deletion and translocation that cause loss of Can1 function) compared to the *lys2–10A* assay which detects single nucleotide deletion mainly within a run of A or T. Combining *elg1*Δ with *msh2*Δ did cause a synergistic increase in mutation rate in the *CAN1* inactivation assay (Figure 3C), in contrast to our finding at the *lys2–10A* assay. Sequencing *can1* from canavanine resistant mutants confirmed the majority of mutations to be single nucleotide deletions or substitutions, as opposed to large deletions or translocations (Supplementary Figure S5), despite the fact that a single *elg1*Δ mutant exhibits elevated recombination levels (15–17). At this locus deletion of *ELG1* may therefore affect MMR-independent repair or stimulate nucleotide misincorporation to increase the mutation rate. In the dissociation-prone mutant *pol30-R14E* and the double *msh2*Δ *pol30-R14E* backgrounds, deletion of *ELG1* did not increase the mutation rate in the *CAN1* assay (Figure 3C), suggesting that PCNA accumulation on DNA in *elg1*Δ remains the cause of increased mutation rate, affecting MMR-independent process(es). Note that we do not know if MMR is also affected by PCNA accumulation on DNA in the *CAN1* locus in our experimental condition.

Davidson *et al.* previously reported that the mutator phenotype of the *elg1*Δ single mutant and the *elg1*Δ *pol30-flag* strain observed at the *CAN1* locus was attenuated by deletion of the transcriptional factor *SWI4*, which mediates expansion of dNTP pools (62). They proposed that increased dNTP levels are correlate with increased mutagenesis (62). We test if deletion of *SWI4* also attenuates the mutator phenotype of the *elg1*Δ mutant observed in *lys2–10A* locus. In the *swi4*Δ background, deletion of *ELG1* still increased the mutation rate at the *lys2–10A* locus, although deletion of *SWI4* reduced the mutation rates both in *ELG1*^+^ and *elg1*Δ (Supplementary Figure S4C). This result suggests that while dNTP pools might affect the mutation rate at the *lys2–10A*, the increased mutation rate caused by deletion of *ELG1* at the *lys2–10A* is not due to expansion of dNTP pools. In contrast to the *lys2–10A* locus, the increased mutation rate in *elg1*Δ at the *CAN1* locus is suppressed by deleting *SWI4* (62), consistent with the idea that the downstream effect of PCNA accumulation on DNA differs at the *lys2–10A* and the *CAN1* loci. In this study we focus our further analyses on the effect of PCNA accumulation on MMR-dependent pathway at the *lys2–10A* locus.

### Over-retained PCNA hyper-recruits the MutS homolog to chromatin through its PIP motif, affecting MMR

We next tested how PCNA over-accumulation on DNA perturbs Msh2-dependent mismatch repair. We envisaged three potential scenarios: (i) over-retained PCNA acts as obstacle and prevents Msh2–Msh6 from sliding along DNA as it scans for mismatches, (ii) over-retained PCNA causes inappropriate recruitment of the MutS homolog (via its PIP-box motif) to DNA where there is no mismatch or (iii) over-retained PCNA prevents mismatch repair indirectly through inappropriate nucleosome deposition by the PIP-box-containing histone chaperone CAF-1.

**Figure 4.**
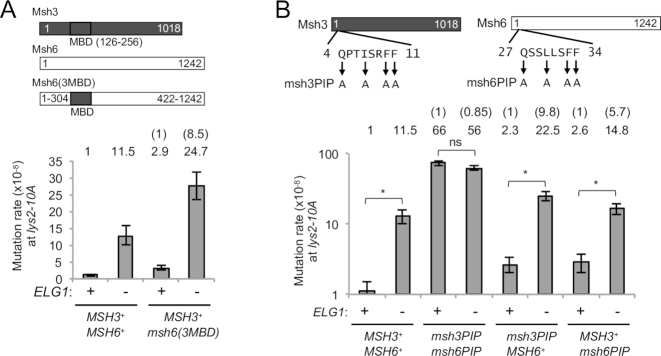
Over-retained PCNA prevents MMR dependently on PIP motifs in the MutS homologs at the *lys2–10A* locus. (**A**) Schematic structure of a chimeric protein Msh6(3MBD) and mutation rate in the *msh6(3MBD)* background at the *lys2–10A* locus. Error bars, 95% confidence intervals. Mutation rates for *ELG1*^+^ and *elg1*Δ are as shown in Figure 3A, repeated for ease of reference here and in subsequent Figures 4B and 5I, (**B**) Mutation rates in the *msh3PIP* and *msh6PIP* mutants backgrounds at the *lys2–10A* locus. The mutation sites in the PIP motifs are shown. Error bars, 95% confidence intervals. *, no overlapping of error bars; ns, overlapping of error bars.

Single-molecule analysis using DNA curtains has shown that the Msh2–Msh3 complex can ‘hop over’ obstacles on DNA, while Msh2–Msh6 complex cannot (63). A Msh6 chimera with its mispair-binding domain (MBD) replaced by that of Msh3 hops over obstacles on DNA (63,64). However, in this chimera *msh6(3MBD)* background, we observe that deletion of *ELG1* still caused an ∼8.5-fold increase in mutation rate (Figure 4A). This result makes it unlikely that prevention of Msh2–Msh6 sliding along DNA by accumulated PCNA is the primary cause of mutation rate increase in the *elg1*Δ mutant.

We then tested the second scenario that PCNA over-accumulated on DNA over-recruits the MutS homolog to DNA, affecting mismatch repair. To prevent over-recruitment of the MutS homologs to DNA via PCNA, we mutated the PIP-box motifs of Msh3 and Msh6. In the Msh3 Msh6 double PIP mutant (*msh3PIP msh6PIP*) background, deletion of *ELG1* did not further increase the mutation rate (Figure 4B), suggesting that over-recruitment of the MutS homologs to DNA through PCNA contributes to the increased mutation rate in *elg1*Δ. Note that the mutation rate of the *msh3PIP msh6PIP* mutant is low enough to test an additive effect (not just a synergistic effect) with *elg1*Δ. In the single *msh3PIP* background where Msh6 can be over-recruited to DNA via PCNA, deletion of *ELG1* caused a 9.8-fold increase in mutation rate, while the single *msh6PIP* background where Msh3 can be over-recruited to DNA caused a 5.7-fold increase (Figure 4B), further evidence that PCNA accumulation on DNA affects the Msh6-dependent pathway to a greater extent than Msh3-dependent pathway. The reason why *elg1*Δ is epistatic with *msh6*Δ (Figure 3A) but not with *msh6PIP* (Figure 4B) might be that Msh3 over-recruited by over-retained PCNA may interfere with the Msh6-dependent pathway where Msh6PIP can be still functional in MMR.

**Figure 5.**
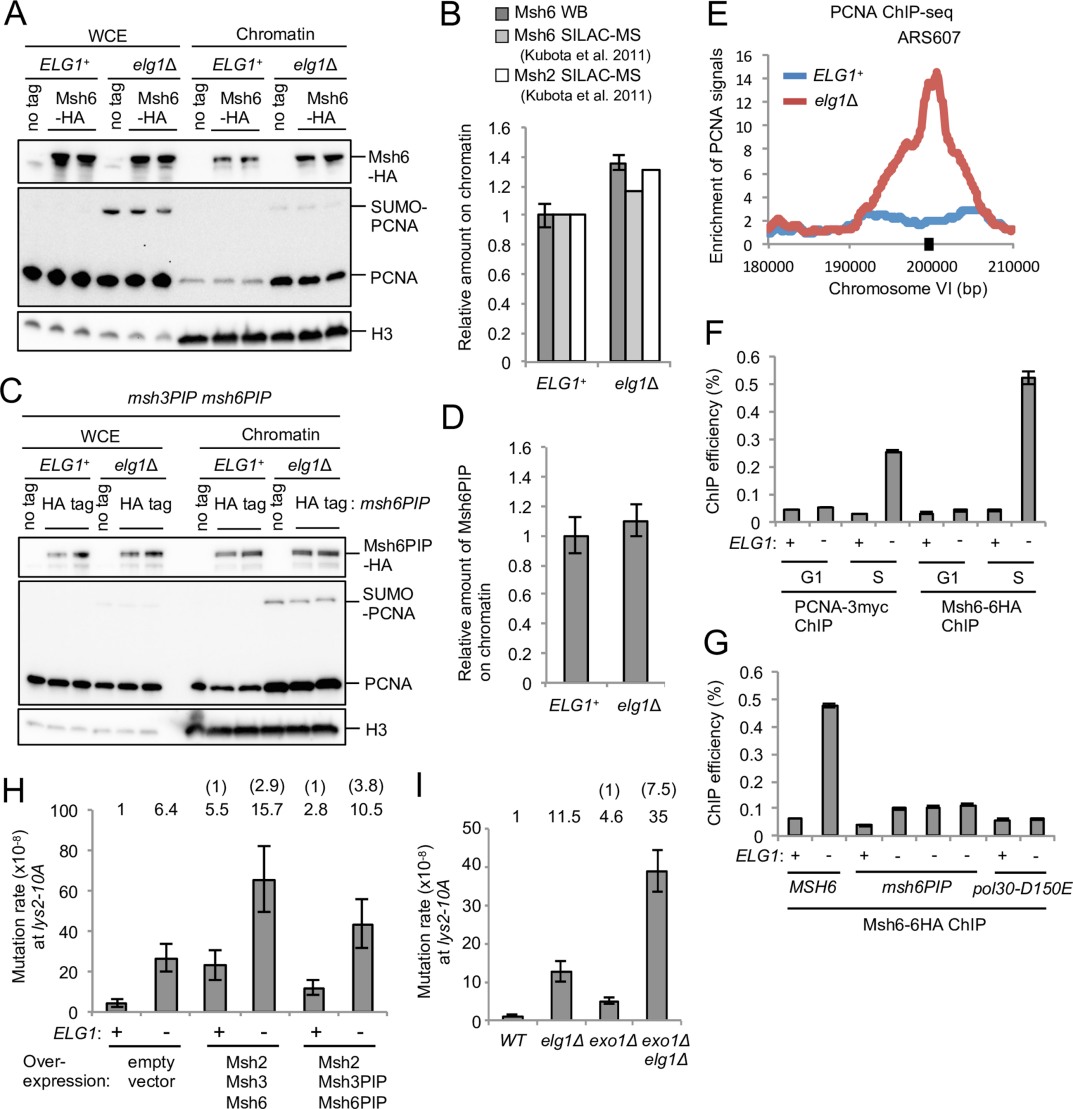
Over-retained PCNA over-recruits Msh2–Msh6 to chromatin, affecting mismatch repair. (**A**) Msh6 accumulates on chromatin in *elg1*Δ, compared to *ELG1*^+^. WCE and chromatin-enriched fractions (Chromatin) were prepared from cells expressing Msh6–6HA in log phase. Msh6–6HA, PCNA and histone H3 (loading control) were detected by western blotting. (**B**) Quantification of chromatin-bound Msh6, normalized to histone H3 (average of two independent clones shown in panel A) and chromatin-bound Msh2 and Msh6 in cells in hydroxyurea by SILAC quantitative proteomics performed previously (46). Error bars, SDs. (**C**) Msh6PIP does not accumulate on chromatin in *elg1*Δ, compared to *ELG1*^+^. WCE and chromatin-enriched fractions (Chromatin) were prepared from *msh6PIP msh3PIP* mutants expressing Msh6PIP-6HA in log phase. Msh6PIP-6HA, PCNA and histone H3 (loading control) were detected by Western blotting. (**D**) Quantification of chromatin-bound Msh6PIP (normalized to histone H3) shown in panel C. Average of two independent clones is shown. Error bars, SDs. (**E**) ChIP-seq analysis of PCNA performed previously (11). PCNA distribution around ARS607 on chromosome VI is shown. PCNA is unloaded behind replication forks in *ELG1*^+^ but retained in *elg1*Δ in S phase (15 min after release from a *cdc7–1* block at 16°C). Black square, region quantified by ChIP-qPCR in panels F and G. (**F**) ChIP-qPCR analysis of Msh6 and PCNA for early origin ARS607 in the presence and absence of Elg1. ChIP was performed using cells arrested in alpha-factor (G1) or collected 15 min after release from a *cdc7–1* block into S phase at 16°C (S). Error bars, SDs of three technical replicates. (**G**) ChIP-qPCR analysis of Msh6 for ARS607 in the *msh6PIP* mutant or the disassembly-prone PCNA mutant *pol30-D150E* backgrounds. ChIP was performed using cells collected 15 min after release from a *cdc7–1* block into S phase at 16°C. Three independent isolates of *msh6PIP elg1Δ* were shown. Error bars, SDs of three technical replicates. (**H**) Effect of over-expression of Msh2, Msh3 and Msh6 or their PIP mutants on mutation rate at the *lys2–10A* locus. Msh2, Msh3 and Msh6 or their PIP mutants are over-expressed under their own promoters from three multicopy plasmids. Error bars, 95% confidence intervals. (**I**) Mutation rates of *ELG1*^+^ and *elg1*Δ in wild-type and *exo1*Δ backgrounds at the *lys2–10A* locus. Error bars, 95% confidence intervals.

We then tested if Msh2–Msh6 is hyper-recruited to over-retained PCNA in *elg1*Δ cells. Western blot analysis of chromatin fractions suggested that Msh6 is over-recruited to chromatin in the *elg1*Δ mutant (Figure 5A and B) and the over-recruitment is dependent on the Msh6 PIP motif (Figure 5C and D). SILAC-based quantitative proteomic analysis has also shown over-recruitment of Msh2 and Msh6 to chromatin in *elg1*Δ in the presence of HU (Figure 5B) (46).

To confirm and further examine over-recruitment of Msh6 to over-retained PCNA in *elg1*Δ at a specific locus, we performed ChIP-qPCR. ChIP-seq analysis previously performed showed that PCNA is generally retained on chromatin in the wake of replication forks in *elg1*Δ (11), including over-retention near ARS607 15 min after release into S phase (Figure 5E), at which point PCNA has already been unloaded in WT cells. To synchronize replication fork movement between cells, cells were released from a *cdc7–1* block into S phase (as performed previously; (11). Consistent with Figure 5E, ChIP-qPCR analysis showed that, in the absence of Elg1, PCNA is over-retained at ARS607 15 min after release into S phase (Figure 5F). We found that Msh6, like PCNA, accumulates at ARS607 15 min after release into S phase in *elg1*Δ (Figure 5F). We observed similar results at ARS305 (Supplementary Figure S6). Over-recruitment of Msh6 to ARS607 in *elg1*Δ largely depends on the Msh6 PIP motif, since signal is greatly reduced in the *msh6PIP* mutant background (Figure 5G). A residual level of Msh6PIP accumulation on DNA in *elg1*Δ suggests some degree of PIP-independence of Msh6 retention when PCNA is over-retained. The over-recruitment of Msh6 to ARS607 in *elg1*Δ is however completely dependent on PCNA over-retention, since in the disassembly-prone *pol30-D150E* background the Msh6 signal in *elg1*Δ is reduced to similar level as in *ELG1*^+^ (Figure 5G). Overall, these results indicate that in an *elg1*Δ mutant Msh6 is hyper-recruited to over-retained PCNA at replicated regions, via its PIP motif. Consistent with our results, knockdown of human Elg1 (ATAD5) also causes PCNA-dependent accumulation of Msh2 on chromatin (13).

Moreover, we observed that over-expression of Msh2, Msh3 and Msh6 from multicopy plasmids caused an increase in mutation rate (Figure 5H, columns 1 and 3) and deletion of *ELG1* results in further increase in mutation rate (Figure 5H, columns 3 and 4), consistent with the idea that over-recruitment of the MutS homolog to chromatin is the cause of the increased mutation rate. Over-expression of Msh2 with Msh3PIP and Msh6PIP has less impact on mutation rates, compared to the corresponding wild-type proteins (Figure 5H, columns 3 and 5). The *elg1*Δ mutation did however increase mutation rate in the context of the PIP mutant protein over-expression (Figure 5H, columns 5 and 6). This increase presumably reflects our observation that the over-expressed PIP mutant proteins do show some accumulation on DNA in *elg1*Δ, as shown in ChIP-qPCR (Figure 5G).

We then tested the third possibility that histone deposition inappropriately competes with MMR through over-recruitment of the CAF-1 histone chaperone. CAF-1 also has a PIP-box motif and accumulates on chromatin in *elg1*Δ (46). However, we did not observe any rescue of the mutator phenotype of *elg1*Δ by deleting a PIP-box-containing CAF-1 subunit (Supplementary Figure S4D and E). Although we cannot entirely exclude the possibility that PCNA accumulation on DNA affects mismatch repair indirectly through other means, our results support the second scenario, in which PCNA over-retained on DNA after replication over-recruits the MutS homolog to DNA through its PIP motif, affecting efficiency of mismatch repair.

### PCNA over-retained on DNA prevents Exo1-independent mismatch repair

After an initial endonuclease cleavage event by the MutL homolog, mispairs are removed by either Exo1-dependent or -independent pathways. PCNA has a central role in the Exo1-independent pathway probably through driving progressive excision by the PCNA-activated MutL homolog endonuclease (35). We tested if PCNA over-retained on DNA in *elg1*Δ prevents Exo1-independent MMR. Deletion of *ELG1* causes further increase in mutation rate when combined with a deletion of *EXO1* (Figure 5I), suggesting that PCNA over-retained on DNA in *elg1*Δ prevents Exo1-independent mismatch repair. Note that we do not know if PCNA over-retained on DNA also prevents Exo1-dependent mismatch repair.

### PCNA over-retained on DNA leads to accumulation of MMR intermediates

**Figure 6.**
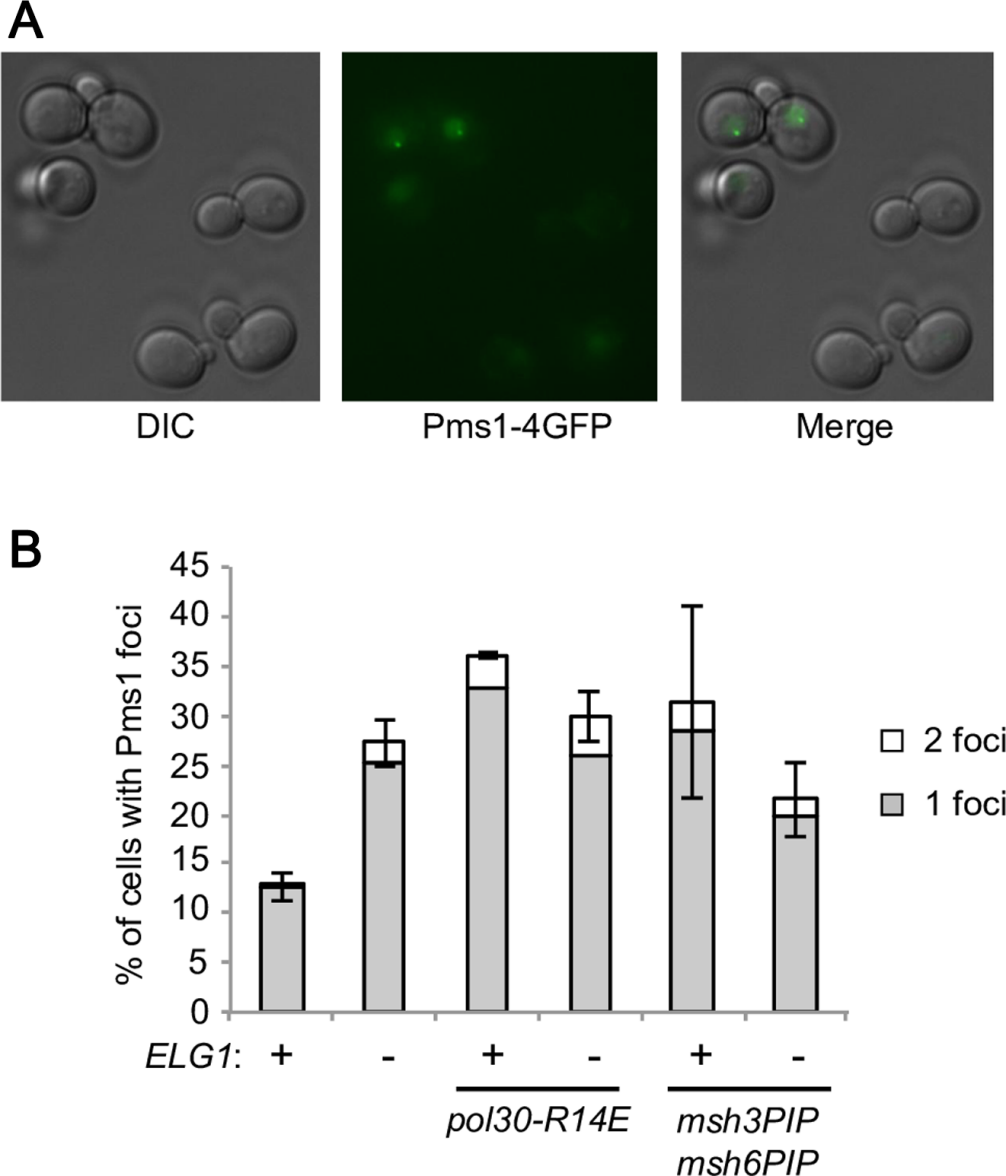
The *elg1*Δ mutant exhibits accumulation of Pms1 foci dependent on PCNA over-retention on DNA and PIP motifs of Msh3 and Msh6. (**A**) Representative image of Pms1-GFP foci. (**B**) Plot shows percentage of cells with Pms1 foci for *ELG1*^+^ and *elg1*Δ in the wild-type and indicated mutant backgrounds. The average value for two independent experiments is presented. Error bars, SDs.

To further test whether deletion of *ELG1* affects MMR, we monitored the recruitment and residence of Mlh1-Pms1 endonuclease, which acts downstream of mismatch recognition by either Msh2–Msh3 or Msh2–Msh6. Cells lacking Elg1 had elevated, rather than reduced, levels of Pms1 foci (Figure 6A and B), indicating that PCNA over-retained on DNA does not prevent recruitment of Mlh1–Pms1. Moreover, we observed accumulation of Pms1 on chromatin in *elg1*Δ by western blot (Supplementary Figure S7). These results suggest that deletion of *ELG1* causes accumulation of Mlh1–Pms1 on DNA.

We next tested if increase of Pms1 foci in *elg1*Δ is caused by PCNA over-retention on DNA. In the dissociation-prone mutant *pol30-R14E* background, deletion of *ELG1* did not further elevate Pms1 foci (Figure 6B), suggesting that accumulation of Pms1 foci in *elg1*Δ is caused by PCNA over-retention. To test if the observed increase of Pms1 foci in *elg1*Δ is caused by over-recruitment of the MutS homologs to over-retained PCNA, we monitored Pms1 foci in *msh3PIP msh6PIP*. In the *msh3PIP msh6PIP* background, deletion of *ELG1* does not change level of Pms1 foci significantly (Figure 6B). These results suggest that accumulation of Pms1 foci in *elg1*Δ is caused by over-recruitment of the MutS homologs via their PIP-box motifs to over-retained PCNA.

It has been previously suggested that Mlh1-Pms1 foci increase when Mlh1-Pms1 is retained longer on mispaired sites due to the defects in the MMR pathway downstream of Mlh1-Pms1 recruitment, and/or when there are more Mlh1-Pms1 recruitment events due to increase in the number of mispaired bases (38). We suspect that increased Pms1 foci in *elg1*Δ is caused by the defects in the MMR pathway downstream of Mlh1-Pms1 recruitment, rather than increase in the number of mispaired bases (if the latter is the case, we would see increase of Pms1 foci caused by deletion of *ELG1* even in the PIP mutant background). We propose that PCNA accumulation on DNA in *elg1*Δ affects the MMR pathway downstream of Mlh1-Pms1 recruitment, causing increase of MMR intermediates.

**Figure 7.**
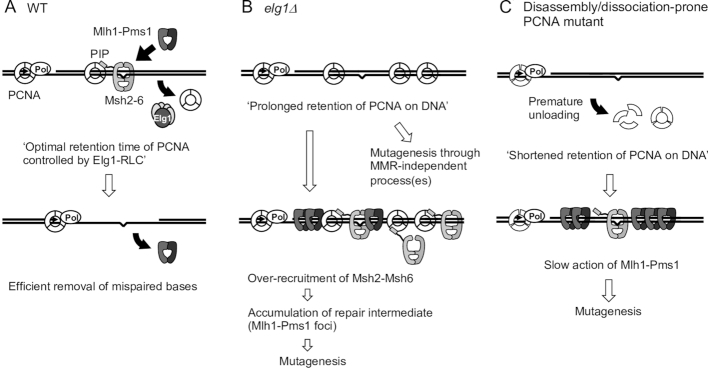
A model for the role of PCNA and Elg1-RLC in mismatch repair and the effect of derailed PCNA regulation on mismatch repair. (**A**) In wild-type, Elg1-RLC unloads free PCNA from DNA after completion of Okazaki fragment processing on the lagging strand, but does not unload PCNA bound to Msh2–Msh6. This will allow factors involved in mismatch repair to work around mispair sites. After removal of mispaired nucleotides by Mlh1-Pms1 and Exo1, Mlh1-Pms1 will dissociate from DNA. (**B**) In *elg1*Δ, PCNA over-accumulates on DNA, leading to over-recruitment of Msh2–Msh6 to chromatin, which in turn, causes accumulation of repair intermediate. PCNA over-accumulation on DNA also affects MMR-independent process(es) to increase mutation rates. (**C**) In disassembly/dissociation-prone PCNA mutants, PCNA will dissociate from DNA prematurely, causing slow or no activation of Mlh1-Pms1 by PCNA.

Taken together, the results shown here suggest that PCNA over-retained behind replication forks in *elg1*Δ over-recruits the MutS homolog to DNA and affects steps downstream of Mlh1-Pms1 recruitment, leading to accumulation of MMR intermediates and a consequent increase in mutation rate (Figure 7).

## DISCUSSION

Cells lacking the PCNA unloader Elg1-RLC exhibit genome instability caused by PCNA over-retained on DNA (24). Cells lacking Elg1 also exhibit a mutator phenotype (26), but it was unknown how deletion of *ELG1* leads to an increased mutation rate or whether PCNA retention time on DNA matters for MMR. In this study, by isolating and analysing PCNA mutants with particular properties, we found that PCNA over-retained on DNA after replication is the cause of the increased mutation rate in *elg1*Δ. Epistasis analysis suggest that PCNA accumulation on DNA interferes with both MMR and MMR-independent processes. We further analyzed the effect of PCNA accumulation on MMR, and found that PCNA over-retained on DNA in *elg1*Δ over-recruits the Msh2–Msh6 complex to DNA and leads to hyper-accumulation of MMR intermediate Mlh1-Pms1 foci. We propose that PCNA over-retained behind replication forks over-recruits the MutS homolog to DNA unnecessarily interfering with downstream steps of mismatch repair (Figure 7). Increased mutation rates in disassembly/dissociation-prone PCNA mutants observed in this study suggest that premature unloading of PCNA also results in increased mutation rates. Taken together, we conclude that proper retention of PCNA on DNA controlled by Elg1-RLC is critical for efficient MMR. PCNA has to be retained on DNA long enough to enable MMR, but if it is retained too long it interferes with downstream repair steps (Figure 7).

How does PCNA accumulation on DNA prevent mismatch repair? In wild-type, Elg1-RLC may unload free PCNA that is not associated with other proteins, so that Msh6-bound PCNA is not unloaded. Interestingly, exemption of Msh6-bound PCNA from unloading has also been proposed based on experiments in the *Xenopus* egg extract system (40) (Figure 7A). This temporary retention of PCNA that is interacting with Msh6 would allow MMR proteins to work properly around mispairs and remove them quickly. In *elg1*Δ, PCNA is over-retained on DNA due to lack of unloading by Elg1-RLC, where we propose it over-recruits PCNA binding proteins including Msh6 (Figure 7B). The over-retained PCNA with the MutS homolog stimulates hyper-accumulation of repair intermediate Mlh1-Pms1 foci, potentially deregulating downstream MMR steps. One possibility is that PCNA bound to the MutS homolog physically occupies the DNA around mispair sites, interfering with the normal rounds of nicking by Mlh1-Pms1 endonuclease (Figure 7B). In addition to the Exo1-independent pathway, PCNA over-retention on DNA might affect the Exo1-dependent pathway by inhibiting the action of Exo1 at steps downstream of Mlh1-Pms1 recruitment. Consistent with our model that hyper-recruitment of Msh2–Msh6 to PCNA is the cause of the increased mutation rate, a very recent study shows co-overexpression of Msh2 and Msh6 increases the mutation rate at *lys2–14A* in a PCNA interaction-dependent manner (as co-overexpression of Msh2 and the Msh6 PIP mutant does not confer a mutator phenotype) (65).

Over-retained PCNA may interfere with other PCNA-related DNA processes such as nucleosome deposition by histone chaperone CAF-1. CAF-1 has a PIP-box motif and accumulates on chromatin in *elg1*Δ (46). Recent work shows that PCNA accumulation causes over-recruitment of CAF-1 to chromatin in *elg1*Δ, leading to defective gene silencing (66). However, the mechanism that disrupts gene silencing differs from that involved in MMR. Janke *et al.* proposed that over-retained PCNA causes the CAF-1 pool to become sequestered from active replication forks in *elg1*Δ, because over-expression of CAF-1 rescues the defect in gene silencing in *elg1*Δ (66). Our study however shows that over-expression of the MutS homologs does not rescue the *elg1*Δ mutator phenotype, but rather increases mutation rates further (Figure 5H). This result suggests that sequestering the MutS homolog is not the cause of increased mutation rate in *elg1*Δ. Hence, over-retained PCNA disrupts both MMR and gene silencing, but through different mechanisms. It remains unknown how over-retained PCNA causes other DNA abnormalities in *elg1*Δ, e.g. increased recombination and telomere elongation.

Does PCNA over-retention affect Msh2-independent process(es) as well as Msh2-dependent MMR? In the *msh2*Δ background, deletion of *ELG1* causes a slight, but significant, increase in mutation rate in the *hom3–10* assays (although not a significant increase in the *lys2–10A* assays) (Figure 3B; Supplementary Figure S4B Table S4). This observation leaves open the possibility that PCNA over-retention prevents Msh2-independent mismatch removal, possibly by base excision repair and/or nucleotide excision repair. In human cells, knockdown of human Elg1 (ATAD5) causes chromatin accumulation of Msh2, together with other replication/repair proteins including FEN1, LIG1 and MRE11 (13), whose over-recruitment might interfere with removal of mispairs. Results of the *CAN1* inactivation assay suggest that PCNA over-accumulation on DNA can increase mutation rate also through MMR-independent process(es) (Figure 3C and B). The observed differences between the *lys2–10A* and the *CAN1* loci in the effects of PCNA over-accumulation probably reflect more frequent slippage events in a long homonucleotide run at the *lys2–10A* locus, where repair largely relies on MMR (47). The *CAN1* inactivation assay in contrast detects many mutation types that can be caused by the defects in MMR-independent repair, such as single nucleotide deletions or base substitutions. These could reflect for example defects in base excision repair or nucleotide excision repair (67), which could potentially also be affected by PCNA over-accumulation. Alternatively, over-accumulation of ubiquitinated PCNA might over-recruit error-prone DNA polymerases to induce mispaired bases at the *CAN1* locus. The mutation pattern (indel vs substitution) in the double *elg1*Δ *msh2*Δ mutant is not clearly different from that in the single *msh2*Δ mutant, but we observed a slight increase of substitution in the double *elg1*Δ *msh2*Δ mutant, compared to the single *msh2*Δ mutant (Supplementary Figure S5). Those substitution occurs mainly at G or C, suggesting that oxidative damage (8-oxo-dG) might be related to those substitutions. How PCNA accumulation on DNA in *elg1*Δ increases spontaneous mutation at the *CAN1* locus remains to be investigated.

All the disassembly/dissociation-prone PCNA mutants tested in this study exhibited increased mutation rate, suggesting that proper retention of PCNA on DNA after DNA replication is important for mismatch repair. When PCNA dissociates from DNA prematurely, it will fail to stimulate MutL homolog endonuclease activity (Figure 7C). We, however, observed substantial variation in the rate of mutation among the dissociation-prone mutants. This may be because the mutants have different abilities to bind the MutS homolog or to stimulate the MutL homolog (35). In addition, the variation may reflect different duration times of dissociation-prone PCNA mutants on DNA, with the mutants that are retained slightly longer on DNA promoting mismatch repair to a greater extent than the mutants that quickly fall off DNA.

In this study, we identified various new dissociation-prone and retention-prone PCNA mutants, providing useful tools to investigate PCNA-related DNA transactions. Previously identified disassembly prone PCNA mutants have mutations at the trimer interface, causing trimer instability as shown by gel filtration analysis of PCNA-C81R and PCNA-E143K (35) and by native gel electrophoresis of PCNA-C81R, PCNA-E113G and PCNA-G178S (61,68). We performed ODN screening and isolated PCNA mutants that fall off DNA spontaneously in *elg1*Δ. As expected, we observed a disassembly-prone phenotype in PCNA mutants that have mutations on the trimer interface (K77E, C81R, R110E, E113K, K117E, K143K, K146E and D150K) (Figure 1 and Supplementary Figure S1C). Interestingly, we observed that mutations on the PCNA inner ring surface also cause a dissociation-prone phenotype (Figure 1 and Supplementary Figure S1C) although at least three of them have previously shown to form stable trimers in *vitro* (35,61). The new inner surface mutants have arginine/lysine to aspartic acid/glutamic acid substitutions: K13E, R14E, K20E and K217E. Crystal structures of yeast and human PCNA revealed that positively charged amino acids on the PCNA inner ring surface are in contact with DNA (51,57). Taken together, these results suggest that while inner surface mutants are able to form stable trimers, they are not well retained on DNA due to the mutations on the inner surface that disrupt electrostatic interactions with DNA. As C22Y does not convert a charged residue it is not in this category, but the crystal structure of PCNA-C22Y revealed that the C22Y substitution alters the alpha-helices that comprise the inner ring, altering the position of DNA-contacting residue K217 (61).

It has been reported that acetylation of lysine residues at the inner surface of PCNA is induced by DNA lesions, and the cohesion acetyltransferase Eco1 targets lysine 20 on the inner surface of the PCNA ring (69). Interestingly, the acetylation-mimicking mutant PCNA-K20Q shows a reduction of PCNA SUMOylated forms (69). This implies that PCNA-K20Q is a dissociation-prone mutant like PCNA-K20E shown in our study. There is the possibility that cells actively control PCNA retention on DNA by acetylating lysine residues at the inner surface of PCNA by Eco1 in response to DNA damage, which would be important for homologous recombination (69).

As well as dissociation-prone PCNA mutants, we identified new PCNA mutants that accumulate on DNA (Figure 2). Retention-prone mutants PCNA-D17K and PCNA-D21K have point mutations on the DNA-interacting region of inner ring. The aspartic acid to lysine substitutions in those PCNA mutants result in enhanced electrostatic interactions between PCNA and DNA, as illustrated by their crystal structures (Figure 2). Our structural analysis and modeling indicates that the D21K variant strengthens direct interactions with the DNA backbone, without a substantial perturbation of the PCNA structure. The PCNA-D21K mutant provides an excellent tool for testing how PCNA retention on DNA affects cellular function.

PCNA association with DNA needs to be spatially and temporally regulated to coordinate the action of many replisome-associated proteins and repair proteins. Our study shows that PCNA retention time controlled by Elg1-RLC is crucial for suppressing spontaneous mutations. Timely regulation of PCNA is important not only for mismatch repair but also other DNA transactions, including recombination, telomere maintenance and gene silencing. Elg1-RLC is the key factor for clearing up the workshop occupied by the functional ‘tool belt’ PCNA protein, and prolonged PCNA residence causes multiple problems in numerous DNA transactions. Both the unloading function of Elg1 and the MMR pathway appear to be conserved in mammals. Knockdown of human Elg1 (ATAD5) causes accumulation not just of PCNA but also the accumulation of other replication proteins on chromatin in a PCNA-dependent manner (13). Over-recruitment of PCNA-interacting proteins may disturb DNA replication and repair, potentially causing the defects observed in ATAD5 knockdown cells, which include chromosome instability and increased spontaneous DNA damage, as well as high tumor incidence in mice with reduced ATAD5 expression (14,22,70). Overexpression of PCNA, Msh2 and Msh6 has been observed in many cancers and is associated with deleterious outcomes and phenotypes (65). Since knockdown of ATAD5 also causes PCNA-dependent accumulation of Msh2 on chromatin (13), its function in MMR may well be conserved in mammals and important for preventing tumor development, a possibility for further investigation.

## DATA AVAILABILITY

Atomic coordinates and structure factors for the reported crystal structures have been deposited with the Protein Data bank under accession number: 6D0R (PCNA-D17K) and 6D0Q (PCNA-D21K).

## Supplementary Material

gkz441_Supplemental_Files
