# Supplementary material for: Effective mismatch repair depends on timely control of PCNA retention on DNA by the Elg1 complex
Source: Nucleic Acids Res. 2019 May 22;47(13):6826–41. doi: 10.1093/nar/gkz441 (PMC6648347; doi:10.1093/nar/gkz441)

Supplementary Information

Effective mismatch repair depends on timely control of PCNA retention on DNA by the Elg1 complex

Lovely Jael Paul Solomon Devakumar, Christl Gaubitz, Victoria Lundblad, Brian A. Kelch and Takashi Kubota

Inventory of Supplementary Information

Supplementary Figure legends

Supplementary References

Supplementary Figure S1. Mutation rate analysis and ODN screening to identify dissociation-prone PCNA mutants
Supplementary Figure S2. ODN screening to identify retention-prone PCNA mutants and their binding activities to Elg1-RLC
Supplementary Figure S3. PCNA variants display higher positive charge in the pore
Supplementary Figure S4. Mutation rates of *ELG1*$^{+}$ and *elg1*Δ in the different deletion mutant backgrounds
Supplementary Figure S5. Mutation pattern at the *CAN1* locus
Supplementary Figure S6. PCNA and Msh6 accumulate behind replication forks around ARS305 in the absence of Elg1.
Supplementary Figure S7. Pms1-GFP accumulates on chromatin in the absence of Elg1.

Supplementary Table S1. Plasmids used in this study
Supplementary Table S2. Yeast strains used in this study
Supplementary Table S3. Oligonucleotides used in this study
Supplementary Table S4. Mutation rates with 95% confidence intervals
Supplementary Table S5. Crystallographic statistics for PCNA-D21K and PCNA-D17K variants

Supplementary Figure legends

Supplementary Figure S1. Mutation rate analysis and ODN screening to identify dissociation-prone PCNA mutants. (A) Mutation rate of *ELG1*$^+$ and *elg1*Δ at the *hom3-10* locus. Fold increases over wild-type are shown above. Error bars, 95% confidence intervals. *, no overlapping of error bars. (B) Sequence results of the *LYS2* gene of the revertants arising in wild type and *elg1*Δ. All reversion events involved the deletion of one nucleotide, mainly a single A in a run of 10 A's within InsE. Δ indicates the position of deletion. 6-bp repeats were highlighted in red. (C) ODN screening to identify dissociation-prone PCNA mutants that failed to accumulate on DNA in *elg1*Δ. Wild-type PCNA (WT) and its mutants were over-expressed from the ADH promoter in multicopy plasmids in *elg1*Δ. Empty vector (Vector) was used as a control. Whole cell extracts were prepared by the alkaline method and analysed by western blot with anti-PCNA antibody. Most PCNA mutants were successfully over-expressed in *elg1*Δ, but some were not (D17K, D21K, D41K, D42K, K127E, E129K, D174K). Reduction of SUMOylated PCNA in whole-cell extract prepared from cells over-expressing PCNA mutants (compared to that from cells over-expressing wild-type PCNA) suggests the possibility that those PCNA mutants fall off DNA. PCNA mutants showing this 'potentially dissociation-prone' phenotype are highlighted in red. *elg1*Δ mutants carrying D17K or D21K plasmids exhibited small (S), mid (M), and large (L) size colonies on the plates. OE, over-expression.

Supplementary Figure S2. ODN screening to identify retention-prone PCNA mutants and their binding activities to Elg1-RLC. (A) ODN screening to identify PCNA mutants that accumulate on DNA even in the presence of Elg1. Wild-type PCNA (WT) and its mutants were over-expressed from the ADH promoter in multicopy plasmids in *ELG1*$^+$. Empty vector (Vector) was used as a control. Whole cell extracts were prepared by the alkaline method and analysed by western blot with anti-PCNA antibody. All PCNA mutants (except for K168E and D174K) were successfully over-expressed in *ELG1*$^+$. A PCNA mutant (D21K) showing accumulation of its SUMOylated forms is highlighted in red. OE, over-expression. (B) A set of PCNA mutants was re-tested whether these mutants show accumulation of SUMOylated forms in whole cell extracts. D17K was chosen because *elg1*Δ mutants carrying the D17K plasmid, like those carrying the D21K plasmid, exhibit smaller colonies and failed to over-express the PCNA mutant (Supplementary Figure S1C), potentially due to their accumulatable phenotype. R61E, D63K and H64E were chosen because these showed a slight accumulation of their SUMOylated forms in panel A. Whole cell extracts were prepared by TCA-based method which would inactivate de-SUMO enzyme Ulp1 rapidly and preserve SUMOylated forms of PCNA. SUMOylated forms of PCNA-D17K and PCNA-D21K increased clearly, compared to those of WT PCNA, while PCNA-R61E, PCNA-D63K and PCNA-H64E showed moderate increases of their SUMOylated forms. Stain-free, total proteins stained on the membrane using stain-free system. (C) Chromatin-bound PCNA and PCNA-D21K during the cell cycle. Cells expressing PCNA (left) or PCNA-D21K (middle) were released from G1

(alpha-factor) into the cell cycle and collected at indicated time points. Whole-cell extracts (WCE) and chromatin-enriched fractions (Chromatin) were prepared and analysed by western blotting. Quantification of chromatin-bound PCNA and PCNA-D21K were shown (right). (D) Interaction between Elg1 and retention-prone PCNA mutants tested by immunoprecipitation. More PCNA-D21K was co-immunoprecipitated with Elg1, compared to wild-type PCNA (left). PCNA-D17K interacts with Elg1 (right).

Supplementary Figure S3. PCNA variants display higher positive charge in the pore. Electrostatic surface potential calculated using APBS (1). Regions near sites of mutation are highlighted with a green circle.

Supplementary Figure S4. Mutation rates of *ELG1*$^+$ and *elg1*Δ in the different deletion mutant backgrounds. Mutation rate of *ELG1*$^+$ and *elg1*Δ in the *msh3*Δ and/or *msh6*Δ backgrounds at *hom3-10* (A), in the *msh2*Δ backgrounds at the *hom3-10* locus, (B), in the *swi4*Δbackgrounds at *lys2-10A* (C), and in the *cac1*Δ backgrounds at *lys2-10A* (D) and at *hom3-10* (E). Mutation rates of *pol2-M644G* and *pol2-M644G msh2*Δ at the *hom3-10* locus are shown in panel B. Error bars, 95% confidence intervals. The number in the brackets, fold changes over *ELG1*$^+$ in each mutant background. *, no overlapping of error bars; ns, overlapping of error bars.

Supplementary Figure S5. Mutation pattern at the *CAN1* locus. (A) Summary of mutation pattern in WT, *elg1*Δ, *msh2*Δ, and *msh2*Δ *elg1*Δ. Genomic DNA from 10 canavanine resistants in each strain were prepared. Loss of the functional *CAN1* gene by translocation/recombination was tested by PCR amplification using primers shown (right). Mutations were identified by sequencing of the PCR products. Indel, insertion or deletion. (B) Mutations identified by sequencing of canavanine resistants. Δ, single nucleotide deletion; dashed line, large deletion.

Supplementary Figure S6. PCNA and Msh6 accumulate behind replication forks around ARS305 in the absence of Elg1. (A) ChIP-seq analysis of PCNA performed previously (2). PCNA distribution around ARS305 on chromosome III is shown. PCNA is unloaded behind replication forks in *ELG1*$^+$ but retained in *elg1*Δ in S phase (15 min after release from a *cdc7-1* block at 16°C). Black square, region quantified by ChIP-qPCR in panel B. (B) ChIP-qPCR analysis of Msh6 and PCNA for early origin ARS305 in the presence and absence of Elg1. ChIP was performed using cells arrested in alpha-factor (G1) or collected at 15 min after release from a *cdc7-1* block into S phase at 16°C (S). Error bars, SDs of three technical replicates.

Supplementary Figure S7. Pms1-GFP accumulates on chromatin in the absence of Elg1. Whole-cell extract (WCE) and chromatin-enriched fractions (Chromatin) were prepared from cells expressing Pms1-GFP in log phase. Pms1-GFP was detected by Western blotting with

anti-GFP antibody. Relative intensity of Pms1-GFP was shown below. Stain-free, total proteins stained on the membrane using stain-free system.

Supplementary References

1. Jurrus, E., Engel, D., Star, K., Monson, K., Brandi, J., Felberg, L.E., Brookes, D.H., Wilson, L., Chen, J., Liles, K. *et al.* (2018) Improvements to the APBS biomolecular solvation software suite. *Protein Science*, **27**, 112-128.

2. Kubota, T., Katou, Y., Nakato, R., Shirahige, K. and Donaldson, A.D. (2015) Replication-Coupled PCNA Unloading by the Elg1 Complex Occurs Genome-wide and Requires Okazaki Fragment Ligation. *Cell Rep*, **12**, 774-787.

Fig S1 (Devakumar)

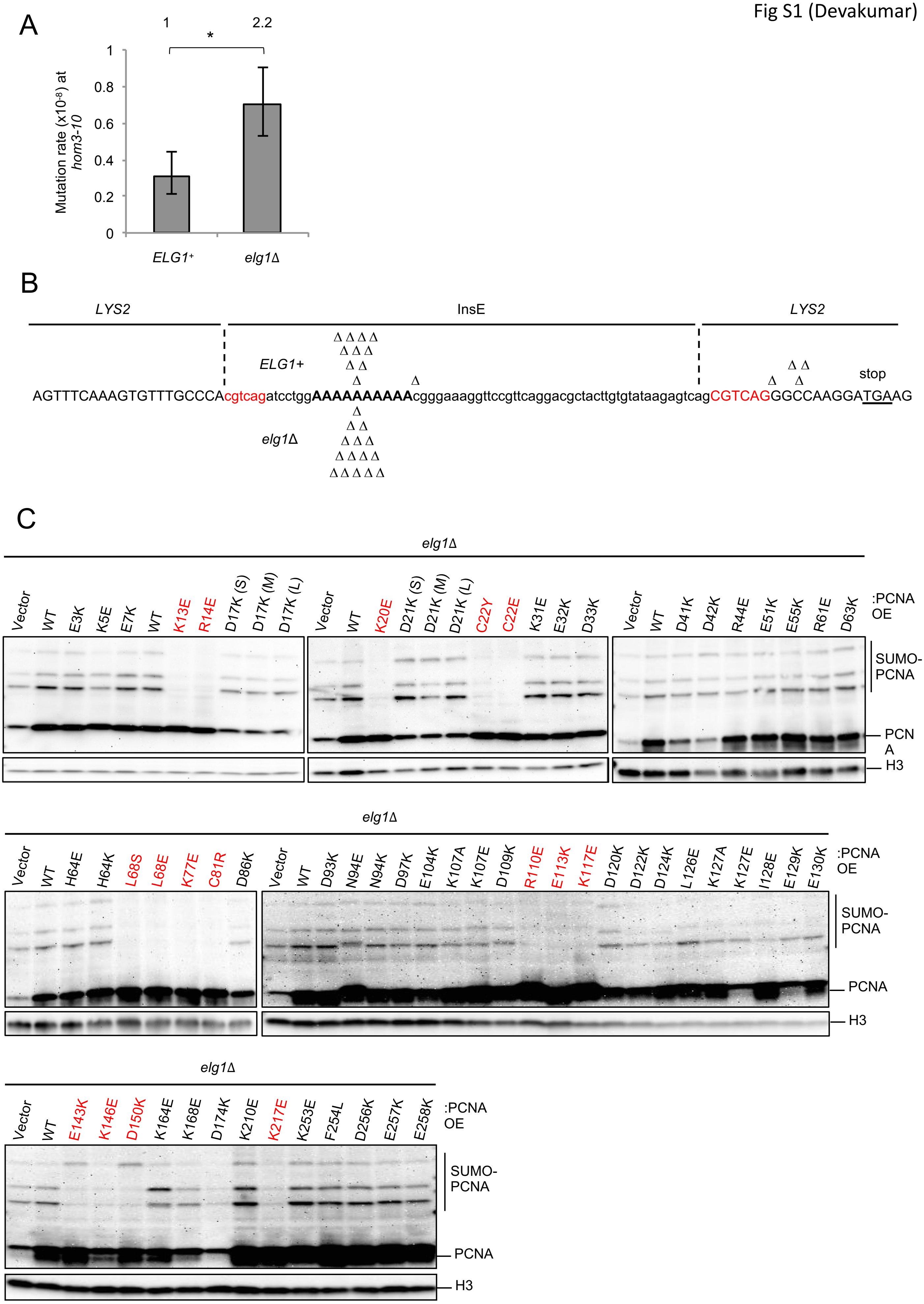

Fig S2 (Devakumar)

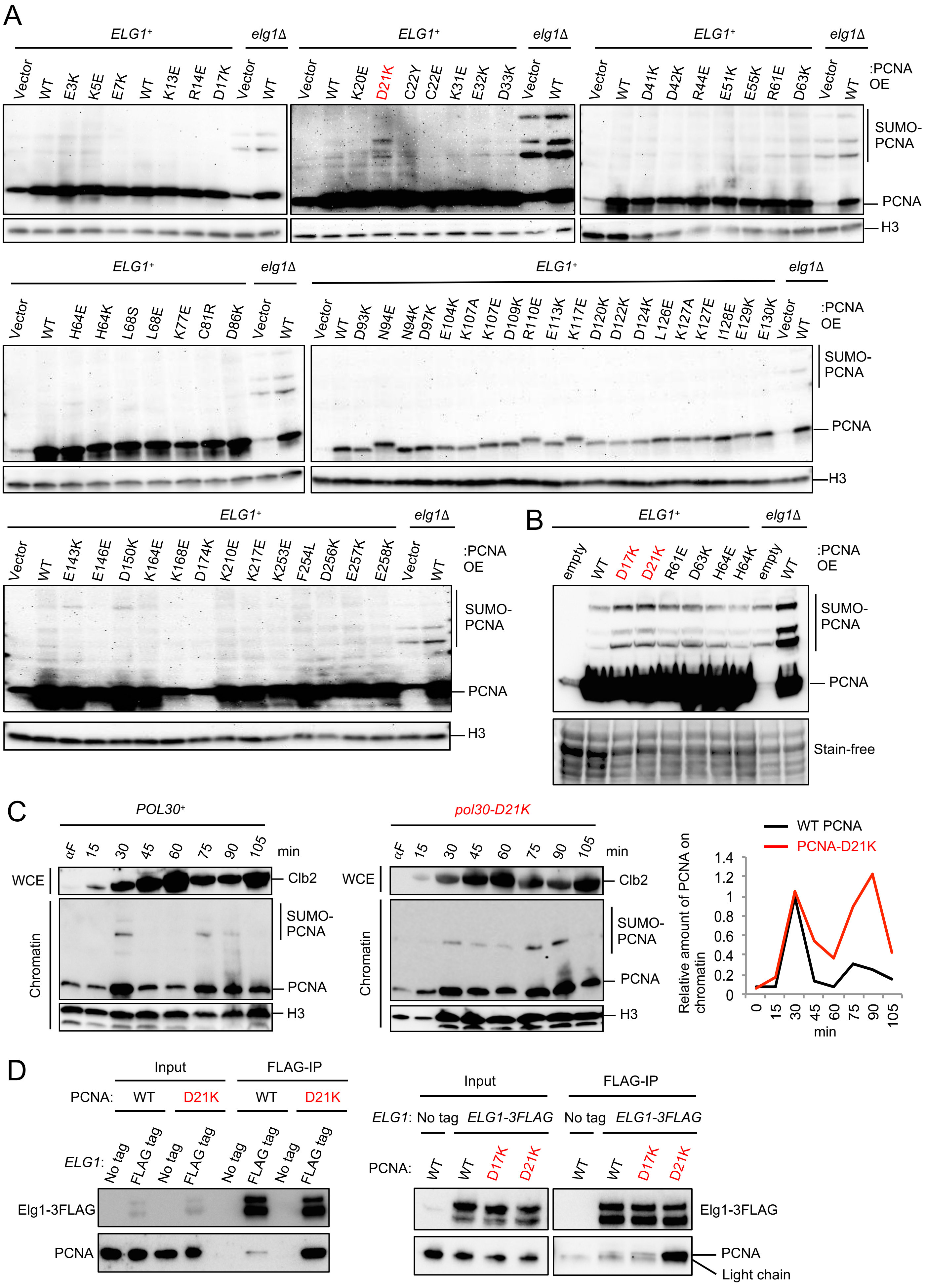

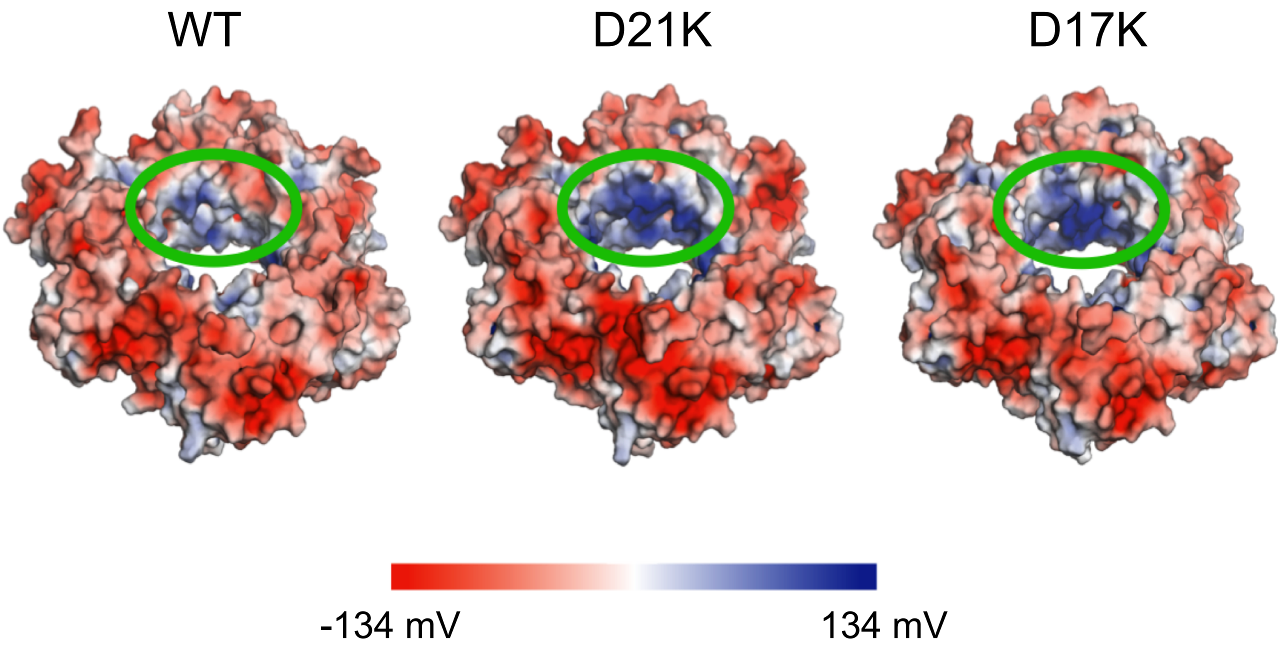
WT
D21K
D17K
-134 mV
134 mV

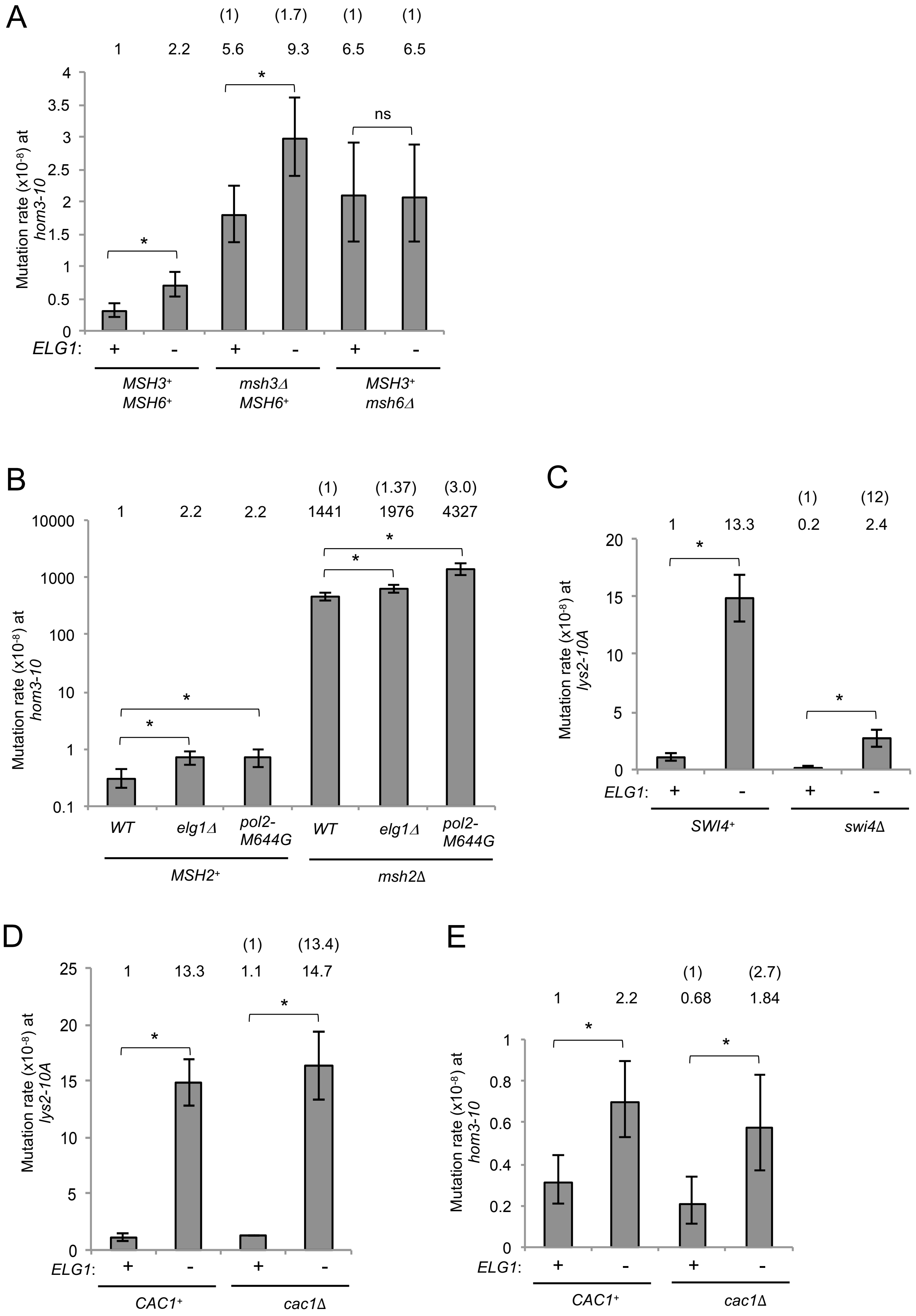
A
(1) (1.7) (1) (1)
1 2.2 5.6 9.3 6.5 6.5
Mutation rate (x10-8) at hom3-10
4
3.5
3
2.5
2
1.5
1
0.5
0
*
*
ns
ELG1: + - + - + -
MSH3+ MSH6+
msh3Δ MSH6+
MSH3+ msh6Δ
B
(1) (1.37) (3.0)
1 2.2 2.2 1441 1976 4327
Mutation rate (x10-8) at hom3-10
10000
1000
100
10
1
0.1
*
*
*
*
WT
elg1Δ
pol2-M644G
WT
elg1Δ
pol2-M644G
MSH2+
msh2Δ
C
(1) (12)
1 13.3 0.2 2.4
Mutation rate (x10-8) at lys2-10A
20
15
10
5
0
*
*
ELG1: + - + -
SWI4+
swi4Δ
D
(1) (13.4)
1 13.3 1.1 14.7
Mutation rate (x10-8) at lys2-10A
25
20
15
10
5
0
*
*
ELG1: + - + -
CAC1+
cac1Δ
E
(1) (2.7)
1 2.2 0.68 1.84
Mutation rate (x10-8) at hom3-10
1
0.8
0.6
0.4
0.2
0
*
*
ELG1: + - + -
CAC1+
cac1Δ

Fig S5 (Devakumar)

A

	Successful PCR amplification	indel	substitution	Substitution at G or C
WT	9/10	2/9	7/9	6/7
*elg1*Δ	9/10	3/8	5/8	5/5
*msh2*Δ	10/10	8/10	2/10	2/2
*msh2*Δ *elg1*Δ	10/10	6/10	4/10	4/4

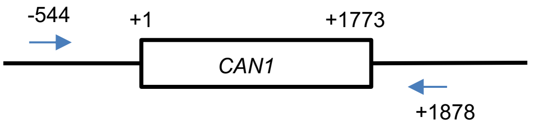

B

Top: WT, red; *elg1*Δ, blue

Bottom: *msh2*Δ, green; *msh2*Δ *elg1*Δ, orange

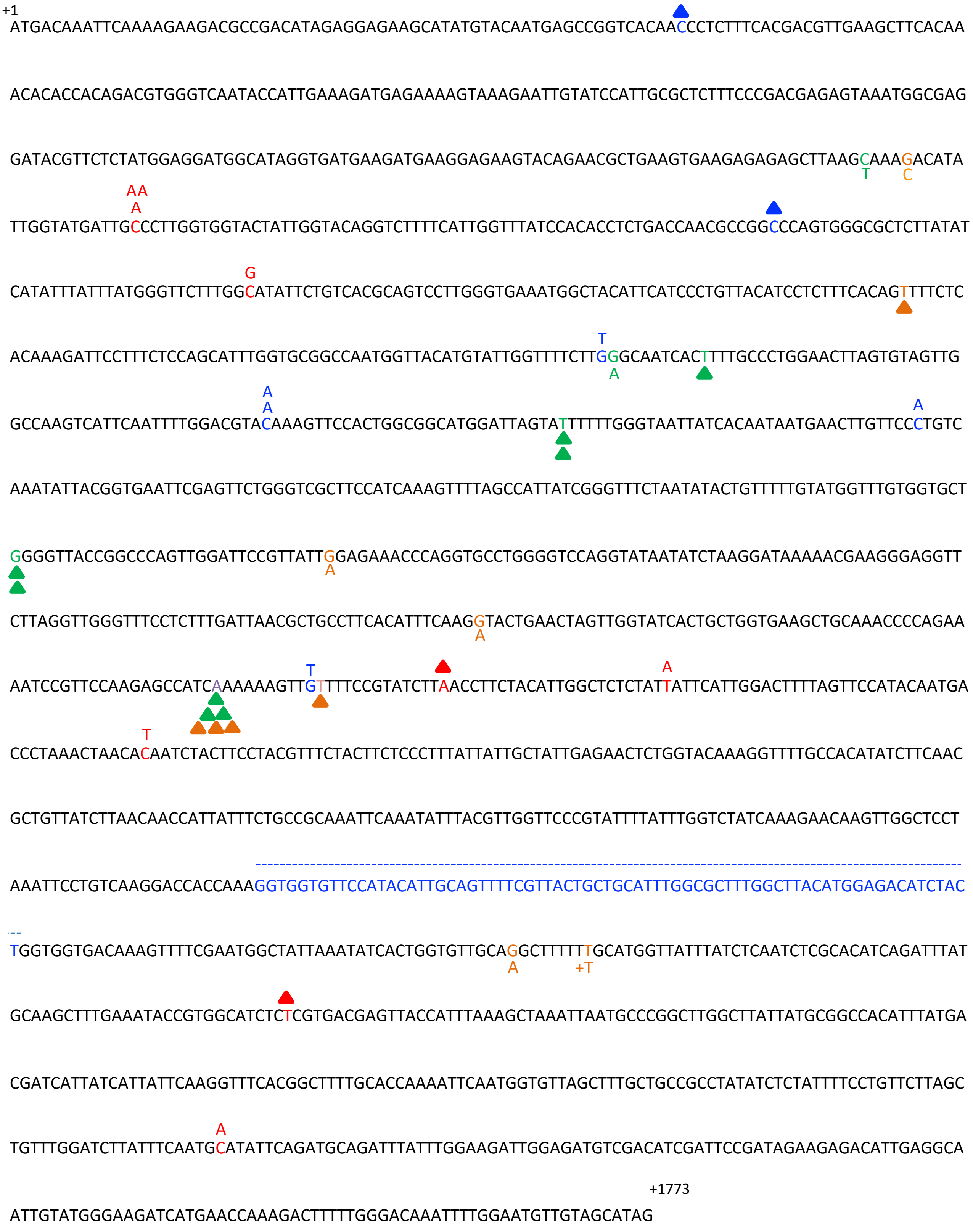

A

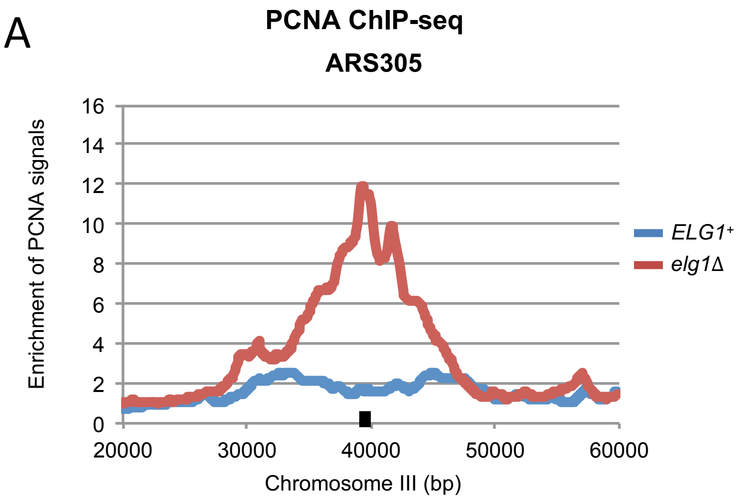
PCNA ChIP-seq
ARS305
Enrichment of PCNA signals
0
2
4
6
8
10
12
14
16
20000
30000
40000
50000
60000
Chromosome III (bp)
ELG1+
elg1Δ

B

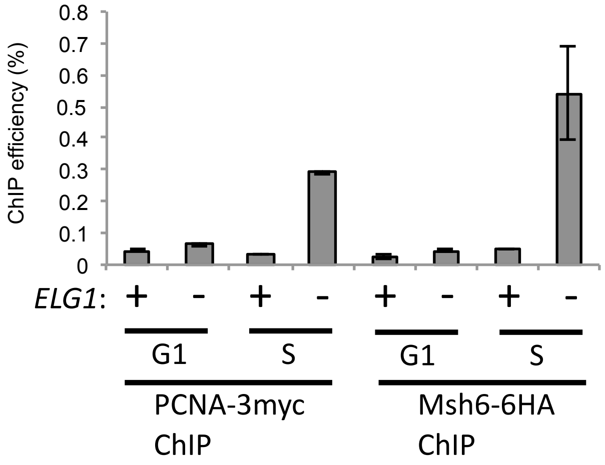
ChIP efficiency (%)
0
0.1
0.2
0.3
0.4
0.5
0.6
0.7
0.8
ELG1:
+
-
+
-
+
-
+
-
G1
S
G1
S
PCNA-3myc ChIP
Msh6-6HA ChIP

Fig S7 (Devakumar)

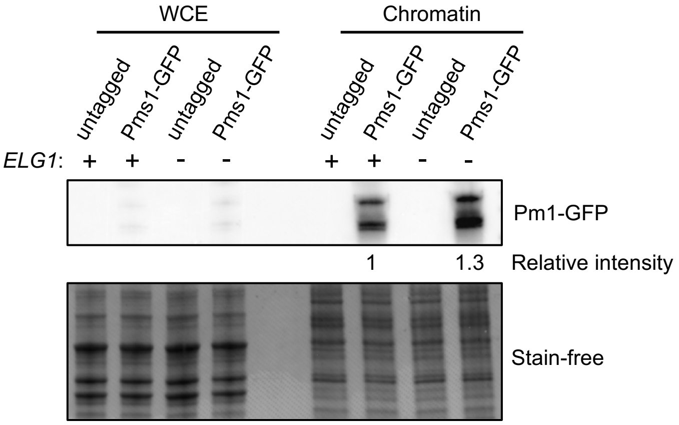

Supplement: gkz441_Supplemental_Files [file gkz441_supplemental_files.zip › Merged_Supple_3May19.pdf]
